# Supplementary material for: Hydrogen bond based smart polymer for highly selective and tunable capture of multiply phosphorylated peptides
Source: Nat Commun. 2017 Sep 6;8:461. doi: 10.1038/s41467-017-00464-0 (PMC5587758; doi:10.1038/s41467-017-00464-0)
Supplement: Supplementary file 1 — Supplementary Information [file 41467_2017_464_MOESM1_ESM.pdf]

**Description of Supplementary Files**

File Name: Supplementary Information
Description: Supplementary Figures, Supplementary Tables, Supplementary Methods and Supplementary References

File Name: Supplementary Data 1
Description: The enriched and identified phosphopeptides with PNI-co-ATBA@SiO2

File Name: Supplementary Data 2
Description: The non-modified peptides identified with PNI-co-ATBA@SiO2, these peptides were co-eluted with phosphopeptides.

File Name: Peer Review File

# Supplementary Methods

## Materials

Silica wafer was purchased from General Research Institute for Nonferrous Metals (China). Amino-modified silica gel (diameter 5 μm, inner pore size 300 Å) was obtained from ACCHROM (A China Chromatography Company, Beijing, China). *N*-isopropylacrylamide (99%, Sigma-Aldrich, abbreviated to NIPAAm) was recrystallized in n-hexane four times before polymerization. Acryloyl chloride (97%), 4-thioureido-benzoic acid, 4-amino-benzoic acid, fluorescein isothiocyanate isomer I (FITC), aminopropyl-trimethoxysilane (ATMS), 2-bromo-2-methylpropionic acid, bromoisobutyryl bromide (BIBB), *N*,*N*,*N*',*N*',*N*''-pentamethyl-diethylenetriamine (PMDETA), aniline, and alanine were purchased from Sigma-Aldrich (St. Louis, MO, USA). A series of mono-, di-, tri-, tetra-serine phosphorylated peptides (1pS—4pS), mono-, di-, tri-threonine or tyrosine PPs (1pT—3pT, 1pY—3pY), five non-modified peptides (e.g., NMP 1–3) used as references, and the corresponding *N*-terminus fluorescein-labeled PPs or NMPs were purchased from China-Peptides Corp. (Shanghai, China) with high purity (> 99.5%). Toluene, acetonitrile ($CH_3CN$), methanol ($CH_3OH$), ethanol, acetone, chloroform ($CHCl_3$), dichloromethane ($CH_2Cl_2$), *N*,*N*'-dimethyl formamide (DMF), dimethylsulfoxide (DMSO), triethylamine ($Et_3N$), ammonium formate ($NH_4FA$) (Alfa Corp., China), and sodium sulphate ($Na_2SO_4$) were used as received, all chemicals used were of chromatographic purity. Double distilled water (18.2 MΩ·cm, MilliQ system, Bedford, MA, USA) was used.

## Biological reagents for PP enrichment experiment

Bovine serum albumin (BSA), α-casein, ammonium bicarbonate ($NH_4HCO_3$), glycolic acid, urea, dl-dithothreitol (DTT), iodoacetamide (IAA), protease inhibitor cocktail (product number: P0044), phosphatase inhibitor cocktail 3 (product number: P8340) and PHOS-Select Iron Affinity Gel were purchased from Sigma-Aldrich (St. Louis, MO, USA). Titanium dioxide ($TiO_2$, particle size: 5 μm, pore size: 100 Å) was ordered from GL Sciences (Tokyo, Japan). Sequencing grade modified trypsin was obtained from Sigma-Aldrich (St. Louis, MO, USA). Trifluoroacetic acid (TFA) was obtained from TEDIA (Fairfield, USA). Acetonitrile

(ACN) was purchased from Merck (Darmstadt, Germany). Formic acid (FA) was bought from Acros Organics (Geel, Belgium). Ammonium hydroxide was purchased from Fluka (Buchs, Switzerland). GE-Loader tips were purchased from Eppendorf (Hamburg, Germany). Ultrapure water was prepared with a Milli-Q system (Millipore, Bedford, MA, USA).

The detailed description for protease inhibitor cocktail and phosphatase inhibitor cocktail 3: The two kinds of inhibitors are supplied as a clear solution in DMSO. Phosphatase initiator cocktail 3 contains individual components with specific inhibitory properties. Cantharidin inhibits protein phosphatase, 2A-(-)-*p*-bromo-levamisole oxalate inhibits L-isoforms of alkaline phosphatases. Calyculin A inhibits protein phosphatases 1 and 2A. While protease inhibitor cocktail contains individual components including AEBSF, Aprotinin, Bestatin, E-64, Leupeptin and Pepstatin A. Each component has specific inhibitory properties. AEBSF and Aprotinin act to inhibit serine proteases, including trypsin, chymotrypsin, and plasmin amongst others. Bestatin inhibits aminopeptidases. E-64 acts against cysteine proteases. Leupeptin acts against both serine and cysteine proteases. Pepstatin A inhibits acid proteases.

## Instruments

Hydrogen and carbon ($^1H$ and $^{13}C$) nuclear magnetic resonance (NMR) spectra were recorded on a Varian Mercury VX 300-MHz and a Varian Inova 600-MHz spectrometer, respectively. Infrared spectra were recorded on a Bruker Vertex 80v fourier transform infrared (FT-IR) spectrometer in combination with platinum-attenuated total reflection (ATR) cell accessory. Mass spectra were obtained with a nano electrospray ionization-quadrupole time-of-flight mass spectrometer (ESI-Q-TOF MS) (Waters, Manchester, UK) or LTQ-Orbitrap Velos coupled with Accela 600 high performance liquid chromatography (HPLC) system (Thermo Corp., CA, USA). Molecular weight of copolymer was measured by gel permeation chromatography (GPC) on a PL-GPC 50 system (Agilent Technologies). X-ray photoelectron spectroscopy (XPS) was obtained with a VG Multilab 2000. Zeta potential was measured on a Malvern Zetasizer 2000/3000 instrument. Ultraviolet and visible (UV-Vis) spectra were recorded on a Shimadzu UV-2550 spectrophotometer. Fluorescence spectra were recorded on a Perkin-Elmer LS55 fluorescence spectrophotometer. Static contact angles were measured by a contact angle measurement system (Dataphysics OCA35

instrument) at ambient atmosphere and a constant temperature of 20 °C. Surface plasmon resonance (SPR) measurement was all carried on a SPR Navi 220A system (Finland). Dynamic adsorption experiments of PPs were conducted on a Q-Sense E4 system (Biolin-Scientific Corp., Stockholm, Sweden). Circular dichroism (CD) spectra were recorded on a Chirascan CD spectrometer (Applied Photophysics, UK). Atomic force microscopy (AFM) investigation was conducted on a flat mica substrate using a Multimode 8 AFM (Bruker, Santa Barbara, USA) in a tapping mode. Scanning electron microscopy (SEM) spectra were recorded on a Hitachi S-4800 SEM (Hitachi Corp., Toyko, Japan). Brunauer-Emmet-Teller (BET) adsorption isotherm curves were recorded on a Micrometrics Tri Star II 3020 nitrogen adsorption-desorption apparatus (Micromeritics Instrument Corp. GA, USA).

## Synthesis and characterization of fluorescein-labeled thioureido-benzoic acid

$Et_3N$ (0.050 g, 0.5 mmol) was added to a solution of 4-amino-benzoic acid (0.137 g, 1 mmol) in 30 mL dry $CHCl_3$, the mixture was stirred for 10 min, then FITC (0.393 g, 1 mmol) was added dropwise to this mixture at ambient temperature, and the mixture was stirred for 24 h (**Supplementary Figure 11**). After that the mixture was washed with water three times, and the organic layer was dried over anhydrous $Na_2SO_4$ overnight. After filtration and evaporation of solvent, the crude product was purified by column chromatography on silica gel, with an elution of $CH_2Cl_2/CH_3OH$ (v/v: 2:1), to obtain the pure product as yellow powder (0.273 g, yield: 52%, m.p. 185 °C). $^1H$ NMR (300MHz, $CD_3OD$): $\delta$ (ppm): 6.52–6.63 (m, 2H, fluorescein-Ph-*H*), 6.67 (s, 2H, fluorescein-Ph-*H*), 6.71, 6.80 (d, d, $J_1 = 9$ Hz, $J_2 = 8.7$ Hz, 2H, fluorescein-Ph-*H*), 7.38 (t, $J_1 = 5.4$ Hz, $J_2 = 8.7$ Hz, 1H, fluorescein-Ph-*H*), 7.50 (t, $J_1 = 9$ Hz, $J_2 = 8.4$ Hz, 2H, fluorescein-Ph-*H*), 7.80 (d, $J = 8.1$ Hz, 1H, Ph-*H*), 7.87 (d, $J = 8.7$ Hz, 1H, Ph-*H*), 7.93-7.97 (m, 2H, Ph-*H*, 2H, CN*H*CS, 2H, Ph-O*H*, 1H, COO*H*); $^{13}C$ NMR (300MHz, $CD_3OD$): $\delta$ (ppm): 65.2, 106.7, 107.2, 115.2, 116.1, 119.9, 126.7, 133.1, 133.8, 134.1, 135.7, 136.1, 145.3, 146.3, 156.6, 157.9, 158.8, 167.6, 170.0, 174.3, 174.7, 176.2; IR: 3212, 2985, 2807, 2505, 1744, 1654, 1589, 1314, 1245, 1173, 1105, 993, 847 $cm^{-1}$; MADLI-MS: m/z calcd. for $C_{28}H_{18}N_2O_7S$: 526.08; found: 527.0292 (M+H). Elemental analysis calcd. (%) for $C_{28}H_{18}N_2O_7S$: C, 63.87; H, 3.45; N, 5.32; S, 6.09. Found: C, 63.72; H, 3.39; N, 5.41; S, 6.00.

## Synthesis of PNI-*co*-ATBA copolymer by atom transfer radical polymerization (ATRP)

A 25 mL round-bottom flask was charged with a solvent mixture containing DMF (0.5 mL), $CH_3OH$ (2.5 mL) and $H_2O$ (2.5 mL), then NIPAAm (1.13 g), ATBA (0.50 g), catalyst copper bromide (CuBr, 0.0142 g), ligand PMDETA (0.0173 g) with relative molar ratios of NIPAAm:ATBA:CuBr:PMDETA = 100:20:1:1 were added to the mixture. The flask was sealed with a rubber septum and evacuated, and back filled with argon four times. After that initiator 2-bromo-2-methylpropionic acid (0.0167 g) was added. The flask placed in a preheated oil bath was maintained at 60 °C for 24 h. Then the reaction mixture was diluted with $CHCl_3$ and passed through a basic alumina column to remove ATRP catalyst. The resulting solution was then concentrated and copolymer was precipitated into excess diethyl ether. The obtained copolymer was dried under vacuum to provide PNI-*co*-ATBA. The number–average molar mass ($M_n$) was 35,500 g·mol$^{-1}$ and poly-dispersity index (PDI) was 1.28, determined by gel permeation chromatography (GPC) on a PL-GPC 50 system, using polystyrenes as standards for calibration and DMF as the eluent at a flow rate of 1.00 mL·min$^{-1}$ at 25 °C.

## Synthesis of PNI-*co*-ATBA thin film on silicon substrate

A clean silicon substrate was immersed in sodium hydroxide (NaOH, 0.1 mol·L$^{-1}$) aqueous solution for 5 min and subsequently in nitric acid ($HNO_3$, 0.1 mol·L$^{-1}$) aqueous solution for 10 min to generate surface hydroxyl groups. After the substrate had been washed with an excess of water and dried under a flow of nitrogen, it was heated to reflux in toluene that contained 5 wt% ATMS for 3 h to obtain chemically bonded amine groups on the surface. The surface was rinsed with dry toluene and dichloromethane to remove remaining ATMS, then dried under a flow of nitrogen gas, and immersed in dry 10 mL dichloromethane that contained pyridine (2% w/v). Then the polymerization initiator bromoisobutyryl bromide (BIBB, 1.0 mL) was added dropwise into the solvent containing the silicon substrate at 0 °C, and the mixture was left for 1 h at this temperature then at room temperature for 12 h. The silicon substrate was cleaned with dichloromethane, and dried under a nitrogen flow. Taking

advantage of a surface initiated atom transfer radical polymerization (SI-ATRP),[1] copolymerization of PNI-*co*-ATBA was achieved by immersing the silicon substrate with the initiator grafted on the surface in a degassed solution of NIPAAm (1.13 g), ATBA (0.50 g, 20 mol% ATBA against NIPAAm) in a degassed mixture of $H_2O$ (5.0 mL), methanol (5.0 mL) and DMF (1.0 mL) containing CuBr (0.0284 g, 0.2 mmol) and PMDETA (0.0346 g) for 6 h, at 60 °C. After that, the reaction was stopped by removing the substrate from the polymerization bath, the copolymer grafted substrate was washed with $CH_3OH$, DMF and $H_2O$, respectively, and dried under a $N_2$ stream. Under this condition, the thickness of the copolymer film was between 18 nm and 20 nm.

## Synthesis of PNI-*co*-ATBA thin film on Au-coated quartz-crystal resonators or SPR sensor chips

Au-coated quartz-crystal (QC) resonator was washed with distilled water and ethanol three times. Then a monolayer of 2-mercaptoethylamine was covered on the gold surface after the Au-coated QC resonator was immersed in a solution of 2-mercaptoethylamine ($1 \times 10^{-2}$ $mol \cdot L^{-1}$) in ethanol for at least 24 h. After that the QC resonator was rinsed with ethanol three times and dried under a flow of nitrogen gas. Then the QC resonator was immersed in dry $CH_2Cl_2$ that contained pyridine (2% w/v). The polymerization initiator BIBB was added dropwise into the solvent containing the QC resonator at 0 °C, and the mixture was left at this temperature for 1 h then at room temperature for 12 h. Subsequently, the QC resonator was cleaned with $CH_2Cl_2$, and dried under a nitrogen flow, generating a bromine-modified Au surface suitable for polymerization. Then the same procedure described above was adopted in order to graft the PNI-*co*-ATBA copolymer thin film on the Au-coated QC resonator.

Similar method was adopted to prepare other reference polymers [i.e., PNIPAAm, PNI-*co*-(phenylcarbamothioyl)-acrylamide (abbreviated to PNI-*co*-PCTA), PNI-*co*-acrylamido-benzoic acid (PNI-*co*-ABA), PNI-*co*-acryloyl-L-alanine (PNI-*co*-AA), poly(ATBA)]-grafted Au-coated QC resonators (see **Supplementary Figure 28** for their chemical structures).

The same method was used to graft PNI-*co*-$ATBA_{0.2}$ on the SPR sensor chip surface.

## $^{1}H$ and $^{13}C$ NMR titration experiments

ATBA is regarded as the critical recognition unit in the smart copolymer. In order to validate the combination between ATBA and phosphates, $^{1}H$ and $^{13}C$ NMR titration experiment were performed to investigate the binding details.[2] To avoid the interference of $D_2O$ with strong suppression effect on hydrogen bonding, $d_6$-DMSO was chosen as the solvent because both ATBA and phosphates were well soluble in it. $HPO_4^{2-}$ anion was prepared according to the reference [3] and tetrabutylammonium ($Bu_4N^+$) works as cation. The detailed preparation method for anion is described as below: The tetrabutylammonium salts were prepared by adding 1 equiv. (for $H_2PO_4^-$, $HCOO^-$, $CH_3COO^-$, or benzene phosphate), 2 equiv. (for $HPO_4^{2-}$), or 3 equiv. (for $PO_4^{3-}$) tetrabutylammonium hydroxide in methanol to a solution of corresponding $H_3PO_4$ (1 equiv.) in dry methanol. The mixture was stirred at room temperature for 2 h and evaporated to dryness under reduced pressure. The resulting syrup was dried at high vacuum for 24 h, checked by NMR and stored in the desiccators. These anion guests will be used in the following NMR titration and fluorescent titration experiment.

Different stoichiometric ratios (0~3) of $HPO_4^{2-}$ were added to the host solution of ATBA monomer (1 $mmol \cdot L^{-1}$) and then the chemical shift changes of active hydrogen protons were recorded and analyzed (see **Figure 1d** in manuscript). In addition, $^{13}C$ NMR (**Supplementary Figure 4**) and two dimensional $^{1}H-^{13}C$ correlation spectroscopy (**Supplementary Figure 5**) were used to investigate the interaction of ATBA with an equimolar ratio of $HPO_4^{2-}$ in $d_6$-DMSO. Similarly, the binding behavior of ATBA with phenyl phosphate anion ($Bu_4N^+$ works as cation)[3] was also studied by $^{1}H$ NMR, as shown in **Supplementary Figure 6**.

## Surface contact angle (CA) measurement

Static contact angle was measured at ambient atmosphere and a constant temperature of 25 °C. A series of $CH_3CN/H_2O$ mixture with different volume ratios ranging from 0:100 to 100:0 were prepared precisely in advance. Before measurement, a silicon substrate grafted with the copolymer film was immersed in the $CH_3CN/H_2O$ mixture for 5 minutes, and then dried under a flow of nitrogen gas. The static CA was recorded for each substrate using the sessile drop method and pure water as a solvent. Each measurement was repeated in triplicated to ensure the reliability of data.

## Polymer lower critical solution temperature (LCST) measurement

The polymer solution was injected into a closed quartz cell and the LCST measurement could be completed within 1 h, under this condition, the solution pH value would not change remarkably. It is worth mentioning that various buffer solutions (e.g., phosphate, Tris-HCl, or ammonium formate) were not used because these buffering agents might also impact on the LCST of the copolymer. Transmittance of copolymer solution at 500 nm was measured by UV-Vis spectrophotometer at different temperature, then the effect of solution pH or addition of PP on the LCST of the copolymer were discussed. According to the dramatic change of transmittance near the LCST, the copolymer LCST was determined to be approximately 28 $^{o}$C in pure water (pH 6.5). Its LCST decreased to 26.6 $^{o}$C at pH 3.0 and increased to 29.1 $^{o}$C at pH 10.0. In addition, upon the additions of PPs, the copolymer LCST decreased from 28 $^{o}$C to 27 $^{o}$C or 26.4 $^{o}$C for 1pS or 4pS, respectively. These data indicated that PNI-*co*-$ATBA_{0.2}$ was a typical thermo-responsive polymer and its LCST was strongly influenced by the solution pH value or the addition of PPs. In this experiment, buffer solution was not used in order to eliminate its effect on the copolymer LCST.

## Fluorescent titration experiment

Fluorescent titration experiment, a typical and widely adopted method for calculating the association constant ($K_a$) in host-guest chemistry,[4] was performed to evaluate the binding affinity of ATBA with various anion guests (i.e., $H_3PO_4$, $H_2PO_4^-$, $HPO_4^{2-}$, $PO_4^{3-}$, $CH_3COO^-$, and $HCOO^-$). For these anions, tetrabutylammonium ($Bu_4N^+$) works as cation. The host fluorescein-labeled thioureido-benzoic acid was prepared as stock solutions in Tris-HCl buffer solution (10 mmol·L$^{-1}$, pH 7.4) for the concentration of $5.0 \times 10^{-6}$ mol·L$^{-1}$. Guest anions were prepared to 0.01 and 0.10 mol·L$^{-1}$ of stock solution in pure water. The work solutions were prepared by adding different volumes of guest solution to a series of test tubes, and then same amount of stock solution of the ATBA host was added into each test tube, followed by dilution to 3.00 mL by Tris-HCl buffer solution. After being shaken for 1 min, work solutions were measured immediately at 20 $^{o}$C using a Perkin-Elmer LS-55 spectrometry. $K_a$ between ATBA

host and anion guest were calculated according to fluorescent intensity changes in the maximum emission peak (**Supplementary Figure 7**).[5] Calculation formula[5,6] and detailed $K_a$ values are shown in **Supplementary Table 1**. Control experiment was also performed in $CH_3CN/H_2O$ (v/v=80:20) Tris-HCl buffer solution (10 mmol·L$^{-1}$, pH=7.4) or in DMSO in order to verify the result of NMR study, as shown in **Supplementary Figure 8 and 9** and **Supplementary Table 2 and 3**.

Furthermore, in order to study the binding affinity between serine PPs (i.e., 1pS—4pS) and ATBA, *N*-terminus fluorescein-labeled PP host was prepared as stock solutions in Tris-HCl buffer solution (10 mmol·L$^{-1}$, pH 7.4) for $2.0 \times 10^{-6}$ mol·L$^{-1}$. ATBA guest was prepared to 0.0175 and 0.175 mol·L$^{-1}$ of stock solution in $H_2O$. The work solutions were prepared by adding different volumes of guest solution to a series of test tubes, and then same amount of stock solution of PP was added into each test tube, followed by dilution to 3.00 mL by Tris-HCl buffer solution. After being shaken for 1 min, the work solutions were measured immediately at 20 $^{o}$C using a Perkin-Elmer LS-55 spectrometry. Association constant ($K_a$) were obtained according to intensity changes in the maximum emission peak. The binding capacities of PPs with ATBA were also investigated in other buffer solutions (i.e., pH 2.0: maleate; pH 3.0: chloroacetate; pH 4.0: formate; pH 5.0 and pH 6.4: pyridine; pH 8.4: Tris-HCl; and pH 10.0: ethanolamine). Detailed $K_a$ values are shown in **Supplementary Table 4**.

## SPR measurement of association rate constant ($K_a$) of peptide on copolymer surface

SPR is an optical phenomenon which is highly sensitive for detecting refractive index changes near the measurement surface, in particular molecular binding or release. Using the SPR phenomenon, multi-parametric SPR is a real-time and label free *in vitro* tool for investigating molecule–molecule interactions, providing information on the kinetics and affinity of the studied system. In this experiment, SPR sensor chips with Au coating of 50 nm were purchased from BioNavis Corp, Finland. The Au-coated sensor chips covered with PNI-*co*-ATBA$_{0.2}$ were prepared through the procedure described above. Initially, the chemical modified chip was washed with DMF and water several times, and then it was put into a

sample chamber for SPR measurement after dried under nitrogen gas. After stabilization of fundamental resonant angle with pure water and buffer solution (80% $CH_3CN/H_2O$ plus 20 mmol·$L^{-1}$ $NH_4FA$, pH=7.4) at 20 °C, PP (i.e., 1pS–4pS) or non-modified peptide (i.e., NMP 1 or 2) buffer solution was pumped into sample chamber by a syringe pump at a speed of 10 μL·$min^{-1}$. The concentrations of these peptides were 200 μg·$mL^{-1}$. During the peptide adsorption process, the resonant angle change was recorded by SPR-Navi software and analyzed by Viewer. Then sensorgrams following the change in resonant angle over time were obtained (**Supplementary Figure 15**). The kinetic evaluation of this SPR data was performed with TraceDrawer software (version 1.4, Ridgeview Instruments AB, Sweden). For NMP 1, 2, 1pS–3pS SPR adsorption data, 1:1 binding mode was applied to fit the data; for 4pS, a 1:2 binding mode was applied to fit the data, then association rate constants ($K_a$) were calculated out according to the fitting curve. Each SPR adsorption experiment was repeated three times to obtain the reliable $K_a$ data.

## Quartz crystal microbalance–dissipation (QCM-D) adsorption experiment

QCM-D adsorption measurement was all carried on a Q-Sense E4 system (Sweden). Au-coated quartz crystals (QC) with intrinsic frequency ($F_0$) of 5 MHz were all purchased from Q-Sense Corp. (Sweden). The Au-coated QCs covered with our copolymers or ATBA monolayer were prepared through the procedure described above. Initially, the copolymer-modified QC was washed with DMF and water three times, and then it was put into a flow-cell for frequency measurement after being dried under nitrogen gas. After stabilization of the fundamental resonance frequency with pure water and the corresponding buffer solution, PP buffer solution was pumped into flow-cell by a peristaltic pump at a constant speed of 0.100 ml·$min^{-1}$. The frequency change was then recorded by Q-Sense software and analyzed by Q-Tools.

In order to verify the rationality of ATBA molecular design, Au-coated QCs covered with PNIPAAm, PNI-*co*-PCTA, PNI-*co*-ABA, PNI-*co*-AA, poly(ATBA) were put into a flow-cell respectively, while the cell temperature was kept at 20 °C. After stabilization of the fundamental resonance frequency with pure water and buffer A (80% $CH_3CN/H_2O$ plus 20 mmol·$L^{-1}$ $NH_4FA$, pH = 7.4), serine tetra-PP (4pS) buffer solution (75 μg·$ml^{-1}$) was then

pumped into flow-cell. We also changed the buffer A with buffer B (80% $CH_3CN/H_2O$ plus 0.2% HCOOH plus 0.1% $NH_4OH$, pH = 5.25) to perform the control experiment. The detailed frequency curves are shown in **Supplementary Figure 29**.

To explore "adsorption conversion window" for PP adsorption, $CH_3CN/H_2O$ mixture with different volume ratios (i.e., 50:50, 60:40, 70:30, 73:27, 75:25, 78:22, 80:20, 85:15, 90:10) were used as the eluent solution with constant pH values of 7.4 at 20 $^oC$. In this work, dynamic adsorption processes of serine tetra-PP (4pS) on the QC surfaces modified with PNI-*co*-ATBA, homo-polymer poly(ATBA) and ATBA monolayer were chosen as examples to display the unique advantage of smart copolymer. Detailed adsorption curves and the corresponding frequency changes at the time of 4000s are shown in **Supplementary Figure 25**. In addition, pH value or temperature of the solution is defined as a single variable, respectively, and a series of control experiments were performed similarly. Detailed experimental data are shown in **Supplementary Figure 26** and **27**.

In order to evaluate the PP adsorption capacity on the mainstreamed $TiO_2$ or $ZrO_2$ surface, we also used $TiO_2$ or $ZrO_2$-coated QC (purchased from Q-Sense Corp.) to perform the control experiment. The optimal loading condition (Buffer A: 80% $CH_3CN/H_2O$ plus 20 mmol·$L^{-1}$ $NH_4FA$, pH = 7.4) was adopted and the cell temperature was maintained at 20 $^oC$. Detailed adsorption curves are shown in **Supplementary Figure 19**. In addition, commercially available $TiO_2$ microspheres (particle size: 5 μm, pore size: 100 Å, ordered from GL Sciences, Tokyo, Japan) were covalently bound onto the QC surfaces (**Supplementary Figure 23a**). Under this condition, serine tetra-PPs (4pS) adsorption-induced frequency change on $TiO_2$ surface was still lower than 180 Hz (**Supplementary Figure 23b**), which was still far smaller than that on our copolymer surface.

## AFM experiment

AFM measurements were performed using a Multimode 8 AFM (Bruker, USA). The Au-coated QC (Sample A) covered with copolymers was prepared through the procedure described above. Then the copolymer-modified QC (Sample B) was treated by a solution of serine mono- (Sample C) or tetra-PP (37.5 μmol·$L^{-1}$ in Tris-HCl buffer, pH 7.4) for 1 hour (Sample D) at 20 $^oC$, respectively. After being dried by nitrogen gas thoroughly, the samples

A-D were all scratched by the syringe needle and AFM images were acquired in a tapping mode under ambient conditions. **Supplementary Figure 36** and **37** show the results of morphology and height images for each sample. Similarly, the copolymer-modified QC was also treated by a solution of serine tetra-PP (37.5 μmol·L$^{-1}$ in $CH_3CN/H_2O$ (v/v=80:20) Tris-HCl buffer solution, pH 7.4) for 1 h. The corresponding AFM images are shown in **Supplementary Figure 38**.

## Circular dichroism (CD) spectroscopy measurement

Model PPs (i.e., 4pS, 1pT, 3pT, 1pY and 3pY) and ATBA were prepared to 0.6 mmol·L$^{-1}$ and 1.2 mmol·L$^{-1}$ of stock solutions in $H_2O$, respectively. The work solutions were prepared by adding different volumes of ATBA solution to a series of test tubes, and then 1 mL stock solution of PP was added into each test tube, followed by dilution to 3.00 mL by $H_2O$. Then the sample was transferred into a cuvette (volume: 3 mL). CD spectra were recorded in a range of 180 to 300 nm at a scan rate of 0.2 nm·s$^{-1}$ at room temperature. Raw data was manipulated by smoothing the curves once and the corresponding CD spectra are shown in **Figure 3d** in manuscript, **Supplementary Figure 44** and **45** in SI.

## Binding investigation by Fourier transform infrared (FT-IR) spectroscopy in Bio-attenuated total reflection (ATR) mode

Infrared spectra were recorded on a Bruker Vertex 80v FT-IR spectrometer with a Bio-ATR cell II accessory, which is based on dual crystal technology: the top crystal is made of silicon, and the second crystal has a hemispherical design and is made of zincselenide (ZnSe). The Bio-ATR II unit is factory-preassigned; hence, no alignment is required. Model PPs and ATBA were prepared to 0.5 mmol·L$^{-1}$ and 1.0 mmol·L$^{-1}$ of stock solutions in $D_2O$, respectively. The samples were prepared by adding 8 μL stock solution of PP to the sample cell, and then different volumes of ATBA solution was added respectively, followed by dilution to 20 μL by $D_2O$. For each measurement, the equipment remained in standby mode for 15 min to ensure the equilibrium of temperature (20 °C) prior to test, and all sample spectra were obtained by 1200 scans by subtracting the $D_2O$ background at a 4 cm$^{-1}$ resolution. Before each measurement, the ATR crystal was cleaned with distilled water and

ethanol carefully and dried sufficiently under nitrogen gas flow.

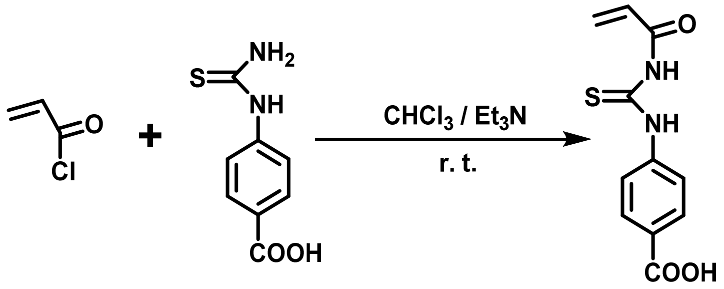


**Supplementary Figure 1 |** Synthesis of 4-(3-acryloylthioureido)-benzoic acid (denoted as **ATBA**)

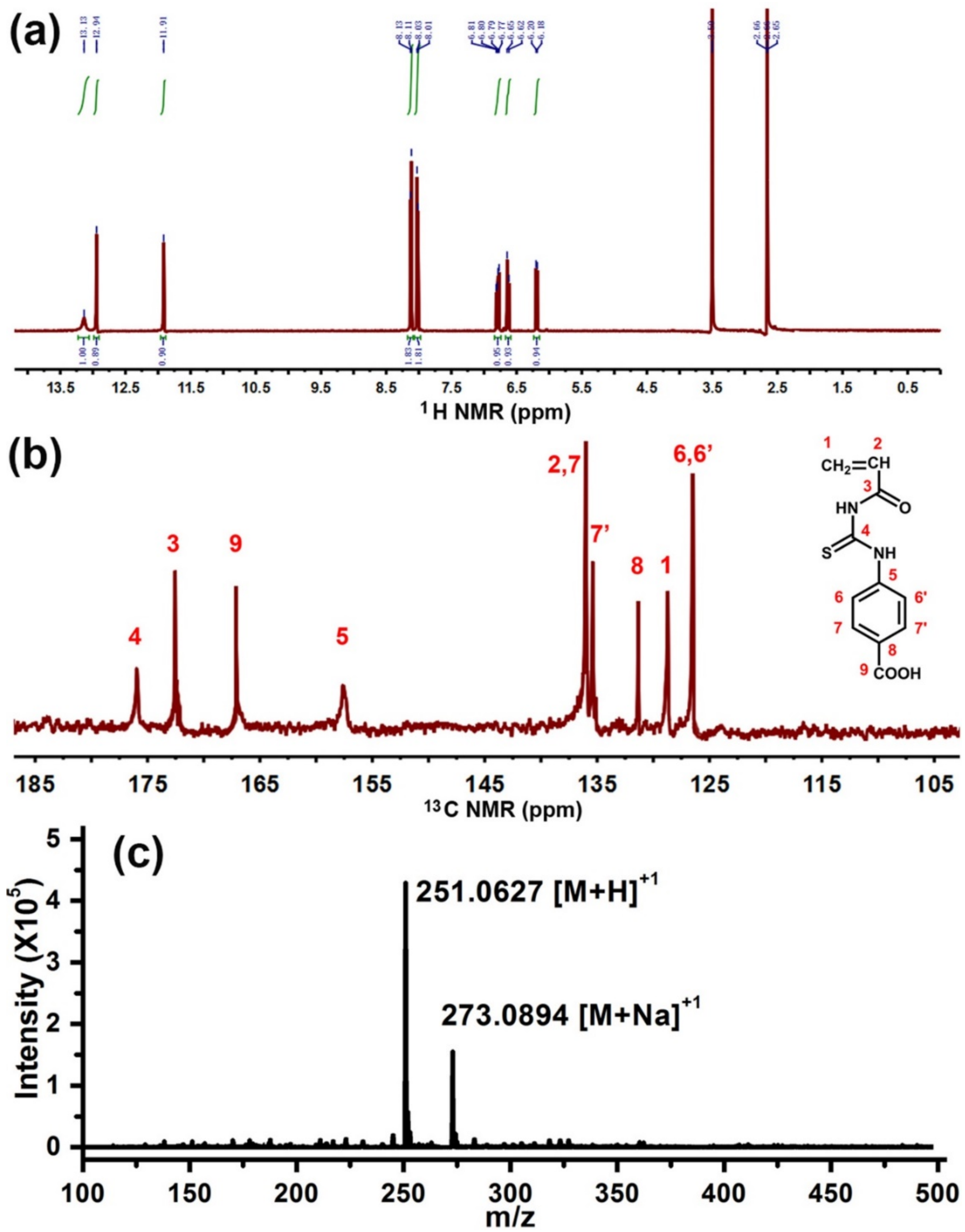


**Supplementary Figure 2 |** $^{1}$H NMR (a), $^{13}$C NMR (b) and MS spectra (c) of ATBA monomer. For NMR experiment, $d_6$-DMSO was used as solvent.

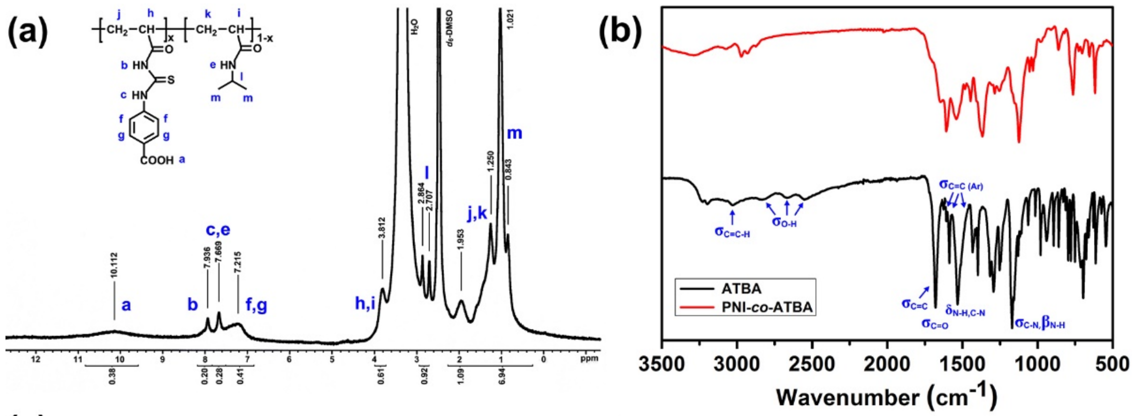


(c)

| Elements | C | H | N | S |
|---|---|---|---|---|
| Content (%) | 60.45 | 8.112 | 8.928 | 3.588 |

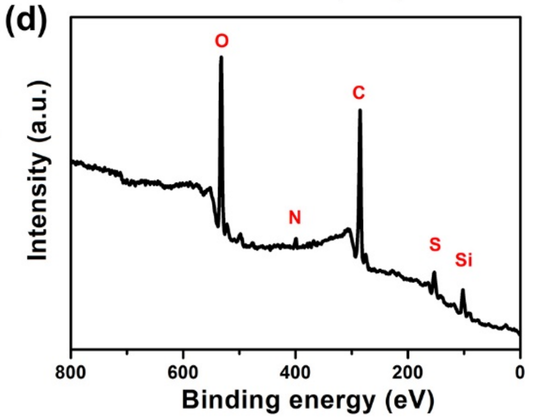


**Supplementary Figure 3 |** Characterization data of PNI-*co*-ATBA$_{0.2}$. (a) $^1$H NMR spectrum of PNI-*co*-ATBA$_{0.2}$ in $d_6$-DMSO at 20 $^o$C, synthesized by ATRP polymerization; (b) FT-IR spectra of ATBA monomer and PNI-*co*-ATBA$_{0.2}$; (c) Elemental analysis data of PNI-*co*-ATBA$_{0.2}$, according to this data, the proportion of ATBA in the copolymer was approximately 20%; (d) XPS spectrum of PNI-*co*-ATBA$_{0.2}$ on silica substrate. The appearance of sulphur element indicated that the copolymer brush had been grafted onto the silica substrate.

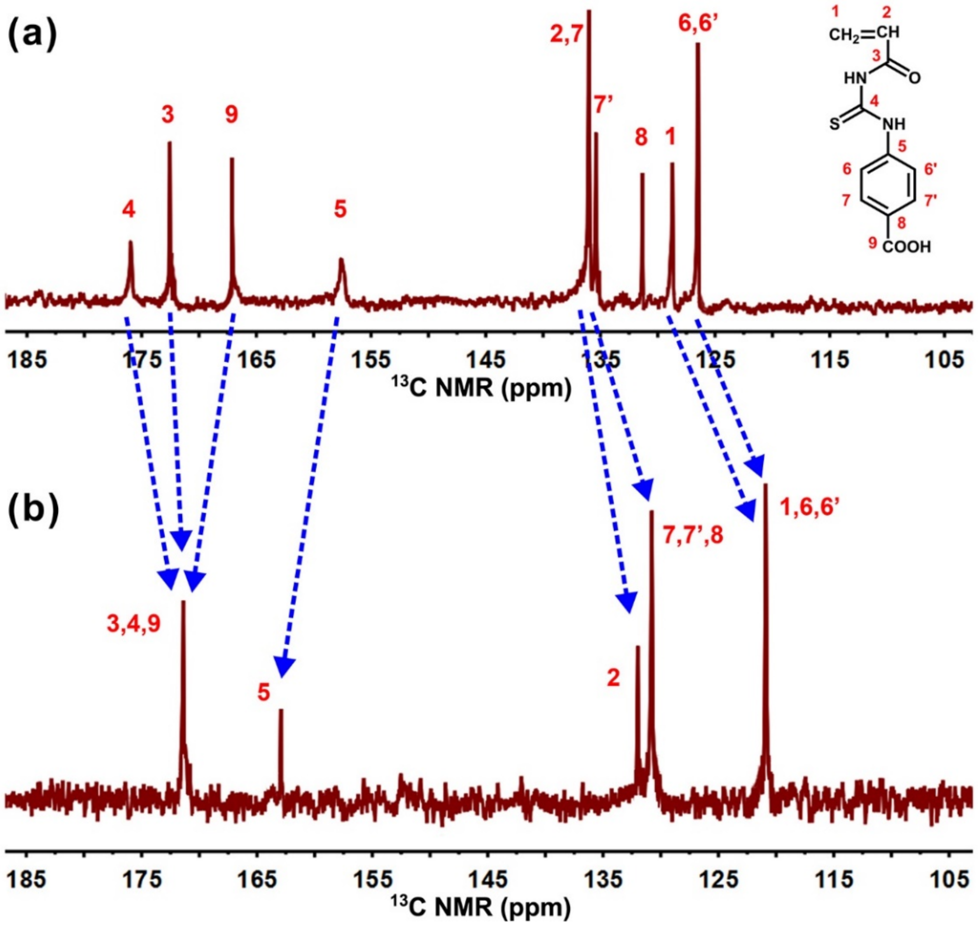


**Supplementary Figure 4 |** $^{13}$C NMR spectra of ATBA before (a) and after (b) interaction with equimolar hydrogen phosphates [$HPO_4^{2-}$, tetrabutylammonium ($Bu_4N^+$) works as cation] in $d_6$–DMSO at 20 °C. (ATBA concentration: $1 \times 10^{-2}$ mol·L$^{-1}$) Chemical shift changes of the ATBA carbon atoms are indicated by blue arrows. As a complement of the $^1$H NMR titration experiment, this data provided another evidence for the complexation between ATBA and $HPO_4^{2-}$ from the perspective of chemical shift changes of carbon atoms in ATBA. We presumed that with the aids of hydrogen bonding interactions between $HPO_4^{2-}$ and thiourea or carboxylic acid group in ATBA, excessively negative charges in $HPO_4^{2-}$ might be transferred to ATBA molecule, resulting in remarkable changes in the chemical shifts of ATBA carbon atoms.

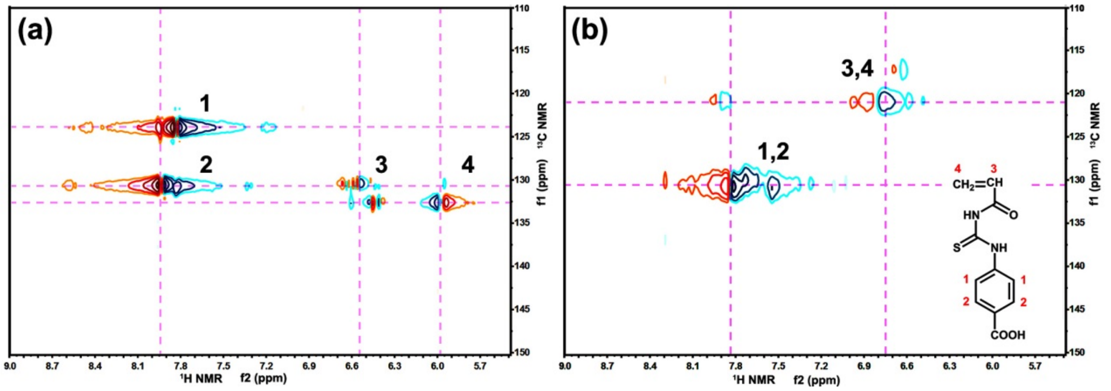


**Supplementary Figure 5 |** $^{1}$H–$^{13}$C correlation spectroscopy of ATBA monomer before (a) and after (b) interaction with equimolar $HPO_4^{2-}$ anion in $d_6$–DMSO at 20 °C. ATBA concentration: $1 \times 10^{-2}$ mol·L$^{-1}$. The 2D NMR spectroscopy further revealed the indispensable role of the unsaturated groups (i.e., phenyl and alkene) in promoting the complexation of ATBA with $HPO_4^{2-}$.

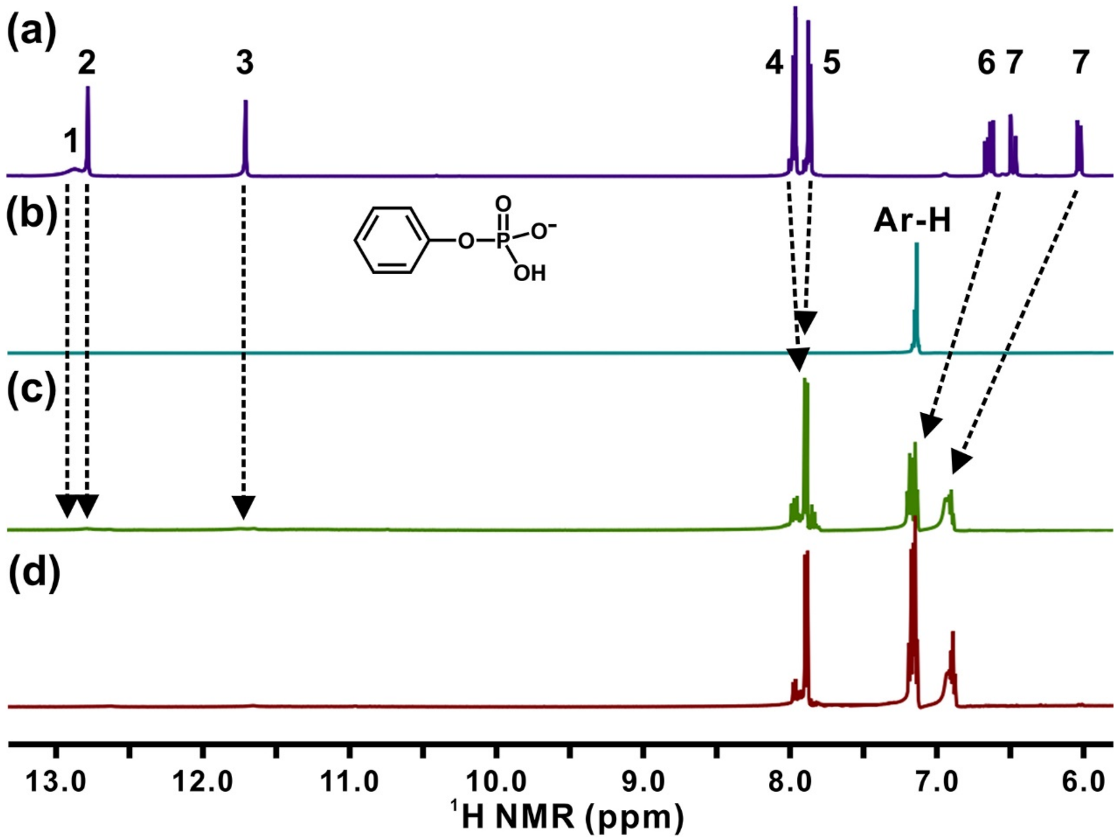


**Supplementary Figure 6 |** $^{1}$H NMR spectra of ATBA monomer before (a) and after (c, d) interaction with different molar ratios of phenyl phosphate (b, $Bu_4N^+$ works as cation) in $d_6$-DMSO at 20 °C. Concentrations: $1.0 \times 10^{-3}$ mol·L$^{-1}$. The molar ratios are 1:0.5 (c) and 1:1 (d). Multiple strong hydrogen bonding interactions were observed for the complexation between ATBA and phenyl phosphate, as indicated by black arrows.

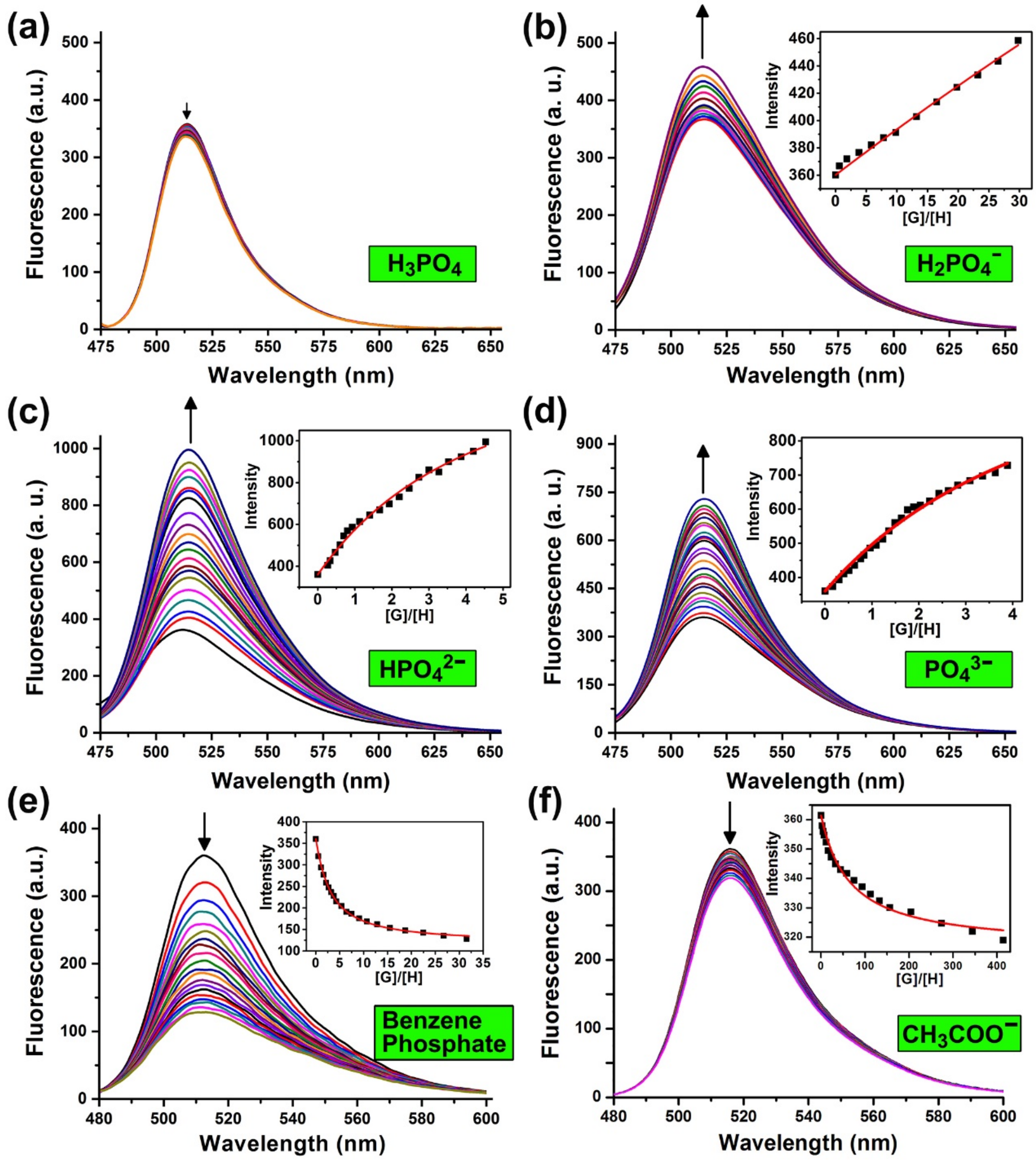


**Supplementary Figure 7 |** Fluorescence spectra of fluorescein-labeled thioureido-benzoic acid ($5.0 \times 10^{-6}$ mol·L$^{-1}$) upon addition of different equivalents of $H_3PO_4$ (a), $H_2PO_4^-$ (b), $HPO_4^{2-}$ (c), $PO_4^{3-}$ (d), benzene phosphate (e) or acetate (f) in Tris-HCl buffer solution (10 mM, pH: 7.4) at 20 °C. For anion guests, tetrabutylammonium ($Bu_4N^+$) work as cations. The insets show the fluorescent intensity changes (at 514 nm) of fluorescein-labeled thioureido-benzoic acid host upon the additions of various anion guests, [G]/[H] is an abbreviation of the molar ratio of guest to host. The red lines are nonlinear-fitted curves. In Tris-HCl buffer solution, the $K_a$ of ATBA with $H_2PO_4^-$, $HPO_4^{2-}$, $PO_4^{3-}$, benzene phosphate or acetate was 500, 53400, 40100, 78000 or 7570 L·mol$^{-1}$, respectively. he $K_a$ ($HPO_4^{2-}$) / $K_a$ ($CH_3COO^-$) ratio was 7.05.

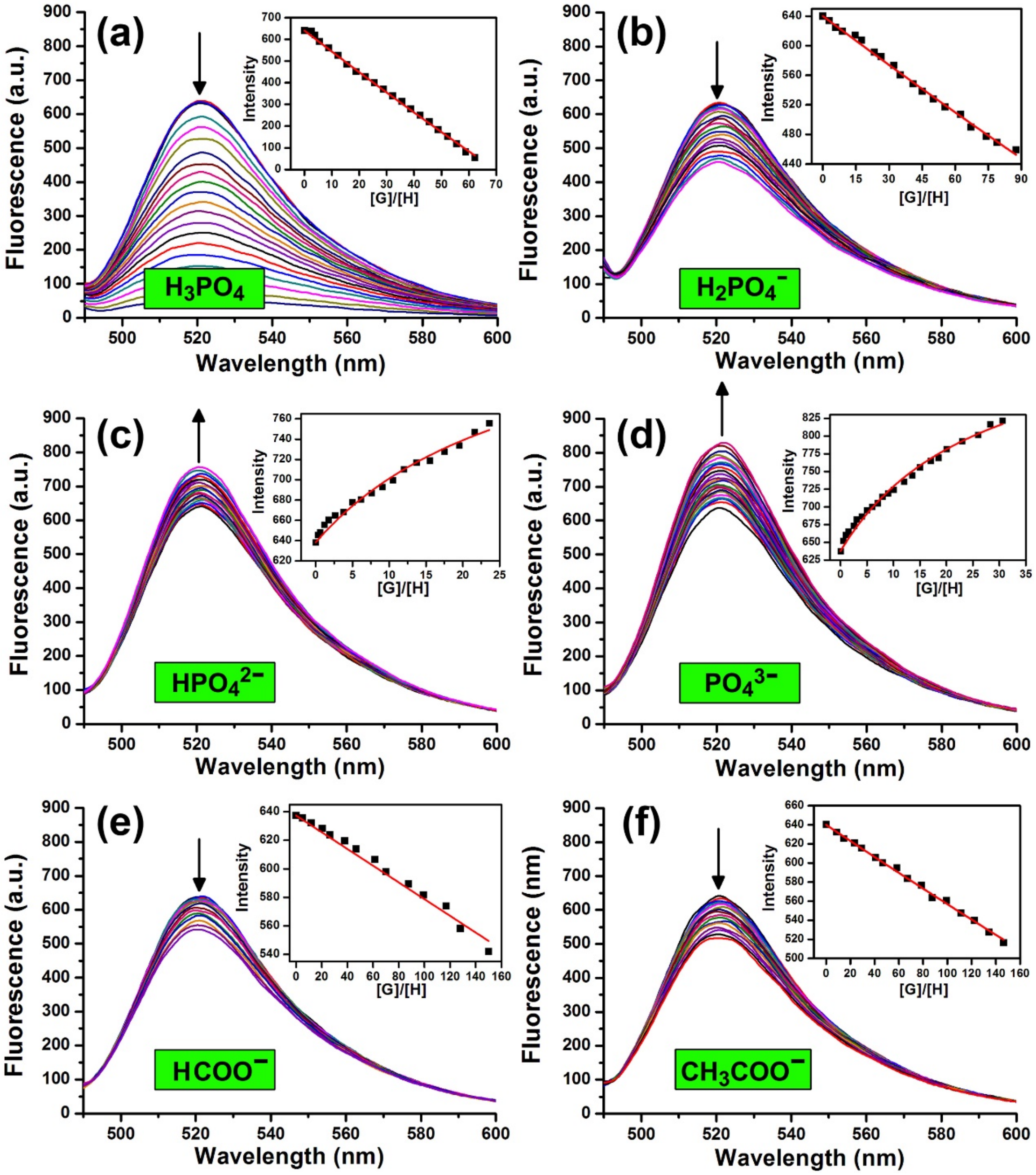


**Supplementary Figure 8 |** Fluorescence spectra of fluorescein-labeled thioureido-benzoic acid ($2.0 \times 10^{-6}$ mol·L$^{-1}$) upon addition of different equivalents of $H_3PO_4$ (a), $H_2PO_4^-$ (b), $HPO_4^{2-}$ (c), $PO_4^{3-}$ (d), benzene phosphate (e) or acetate (f) in $CH_3CN/H_2O$ (v/v: 80:20) Tris-HCl buffer solution (10 mM, pH: 7.4) at 20 °C. For anion guests, tetrabutylammonium ($Bu_4N^+$) work as cations. The insets show the fluorescent intensity changes (at 520 nm) of fluorescein-labeled thioureido-benzoic acid host upon the additions of various anion guests, [G]/[H] is an abbreviation of the molar ratio of guest to host. The red lines are nonlinear-fitted curves. In $CH_3CN/H_2O$ (v/v: 80:20) Tris-HCl buffer solution, the $K_a$ of ATBA with $H_3PO_4$, $H_2PO_4^-$, $HPO_4^{2-}$, $PO_4^{3-}$, formate or acetate was 440, 690, 17500, 19500, 282, or 388 L·mol$^{-1}$, respectively. The $K_a$ ($HPO_4^{2-}$) / $K_a$ ($CH_3COO^-$) ratio was 45.1:1.

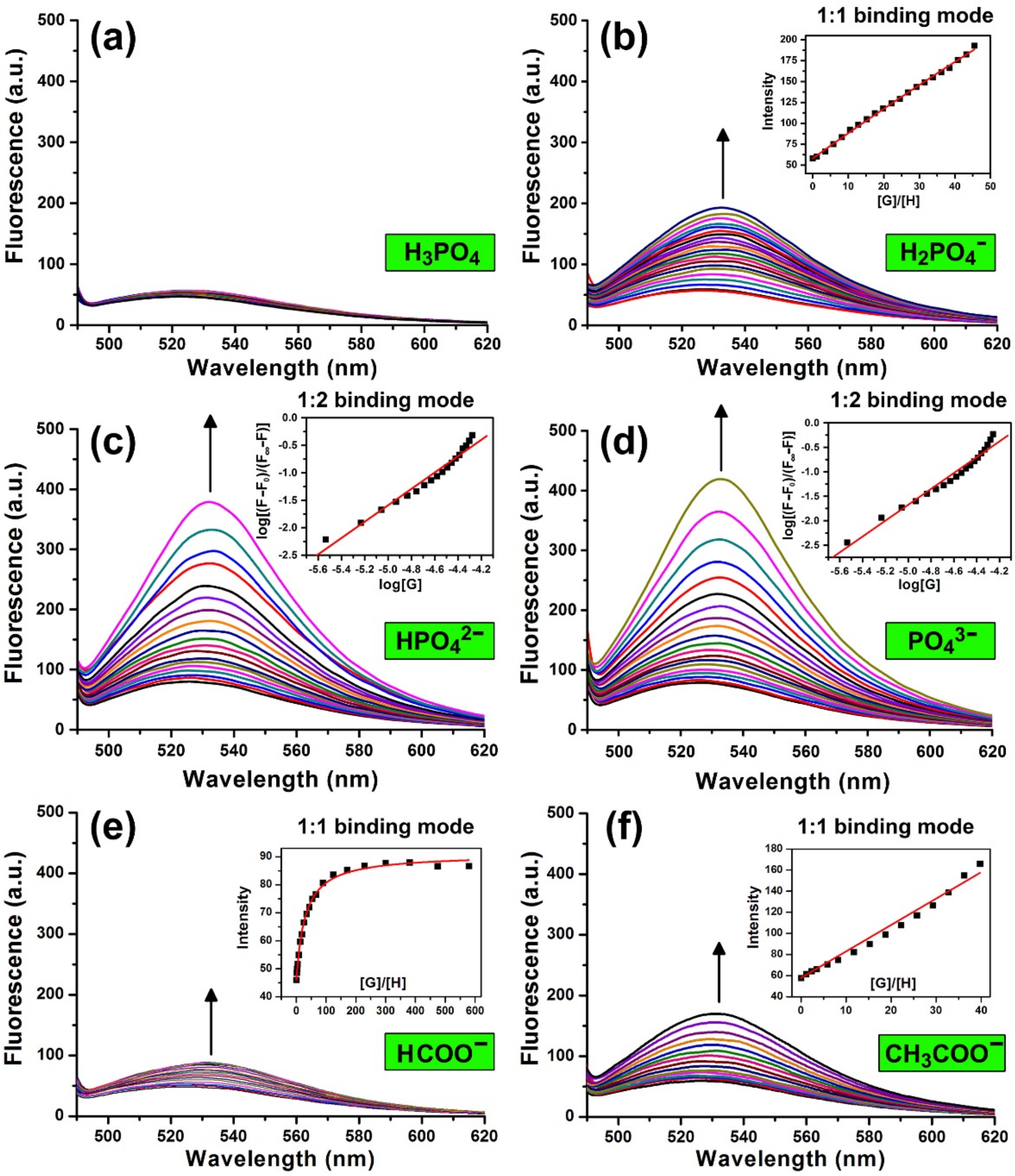


**Supplementary Figure 9 |** Fluorescence spectra of fluorescein-labeled thioureido-benzoic acid ($5.0 \times 10^{-6}$ mol·L$^{-1}$) upon addition of different equivalents of $H_3PO_4$ (a), $H_2PO_4^-$ (b), $HPO_4^{2-}$ (c), $PO_4^{3-}$ (d), formate (e) or acetate (f) in DMSO at 20 °C. For anion guests, tetrabutylammonium ($Bu_4N^+$) work as cations. The insets show fluorescent intensity changes (at 532 nm) of ATBA host upon additions of various anion guests. [G]/[H] is an abbreviation of the molar ratio of guest to host, log[G] is the logarithmic value of guest anion concentration. The red lines are nonlinear or linear-fitted curves. In this experiment, DMSO was chosen as solvent to evaluate the binding affinity of ATBA with various phosphates in polar organic solvent, in which hydrogen bonding interactions were supposed to be substantially stronger than that in aqueous solution owing to the strong suppression effect by water. This presumption was further proven by the $K_a$ data as illustrated in **Supplementary Table 3.** In DMSO, the $K_a$ of ATBA with $H_2PO_4^-$, $HPO_4^{2-}$, $PO_4^{3-}$, benzene phosphate or acetate was 290, $7.14 \times 10^5$, $2.7 \times 10^6$, $6.81 \times 10^3$ or 620 L·mol$^{-1}$, respectively. The $K_a$

($HPO_4^{2-}$) / $K_a$ ($CH_3COO^-$) ratio was 1150:1.

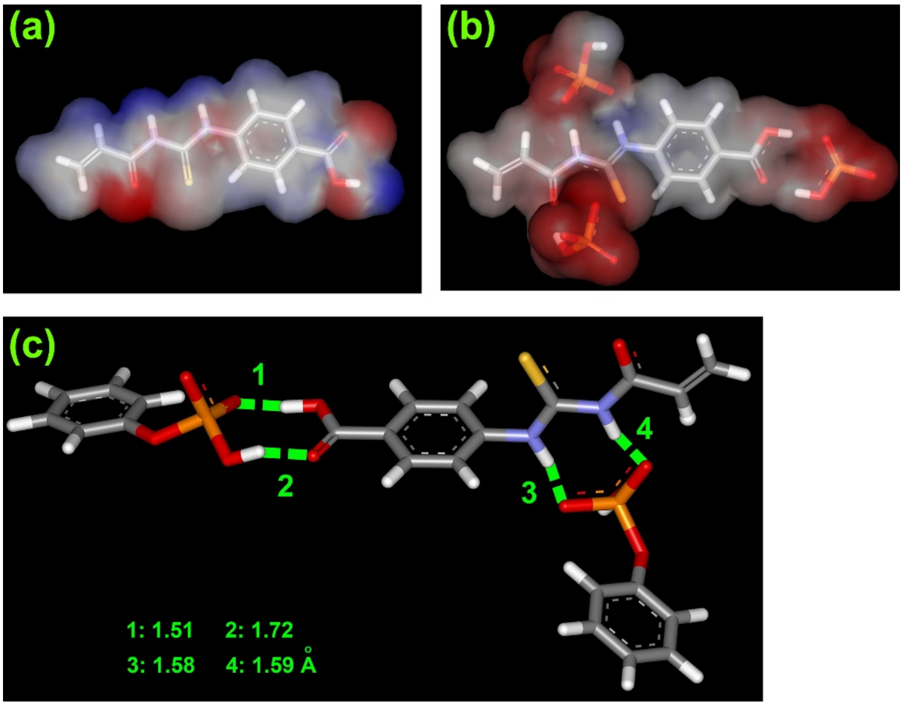


**Supplementary Figure 10 |** Electrostatic potential distribution of ATBA monomer before (a) and after (b) interacting with three $HPO_4^{2-}$ anions, (c) possible binding model of ATBA with two benzene phosphate, obtained from quantum chemistry calculation (Gaussian, density function theory (DFT), at 6-311g level of theory).

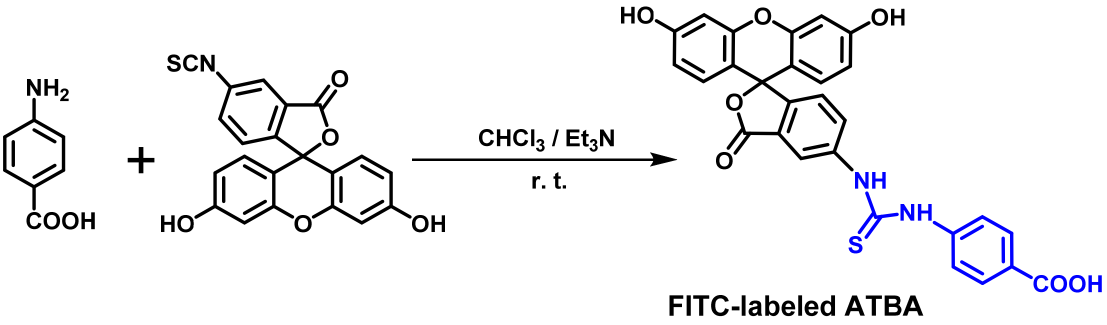


**Supplementary Figure 11 |** Synthesis of fluorescein-labeled thioureido-benzoic acid (chemosensor used in fluorescent titration experiment, it could be regarded as an ATBA analogue.)

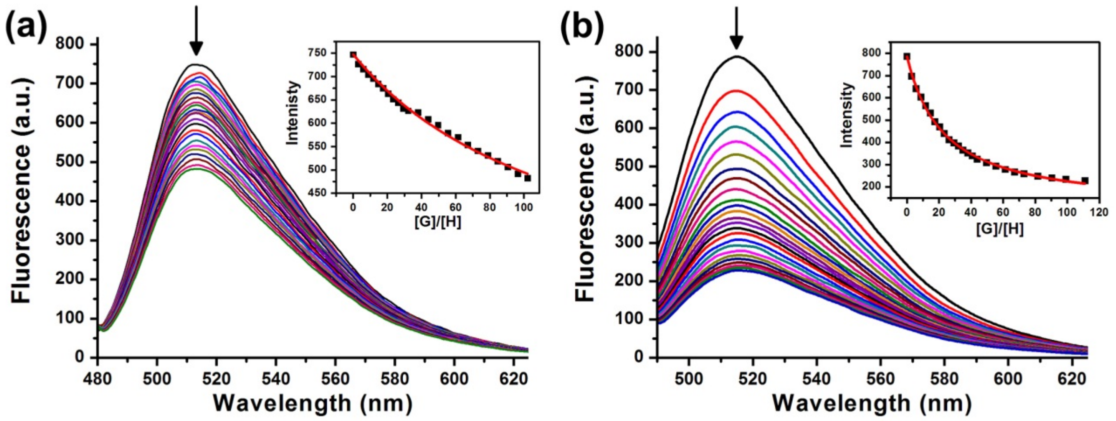


**Supplementary Figure 12 |** Typical fluorescence spectra of *N*-terminus fluorescein-labeled non-modified peptide (e.g., NMP 1) (a) or serine tetra-PP (4pS) (b) ($2.0 \times 10^{-6}$ mol·L$^{-1}$) host upon addition of different equivalents of ATBA monomer guests in formate-buffer solution (pH 4.0, 1 mmol·L$^{-1}$) at 20 °C. The insets show the fluorescent intensity changes of peptide hosts upon the additions of ATBA guest. The red lines are nonlinear-fitted curves. [G]/[H] is an abbreviation of the molar ratio of guest to host. These data indicated that both NMP 1 and 4pS could combine with ATBA, but association constant ($K_a$) of 4pS with ATBA ($K_a$: 22380 L·mol$^{-1}$) was 5.7 times larger than that with NMP 1 ($K_a$: 3940 L·mol$^{-1}$). Through the same method, $K_a$ between ATBA and various PPs were obtained, while the binding capacities at different solution pH were also evaluated, as illustrated in **Supplementary Table 4**.

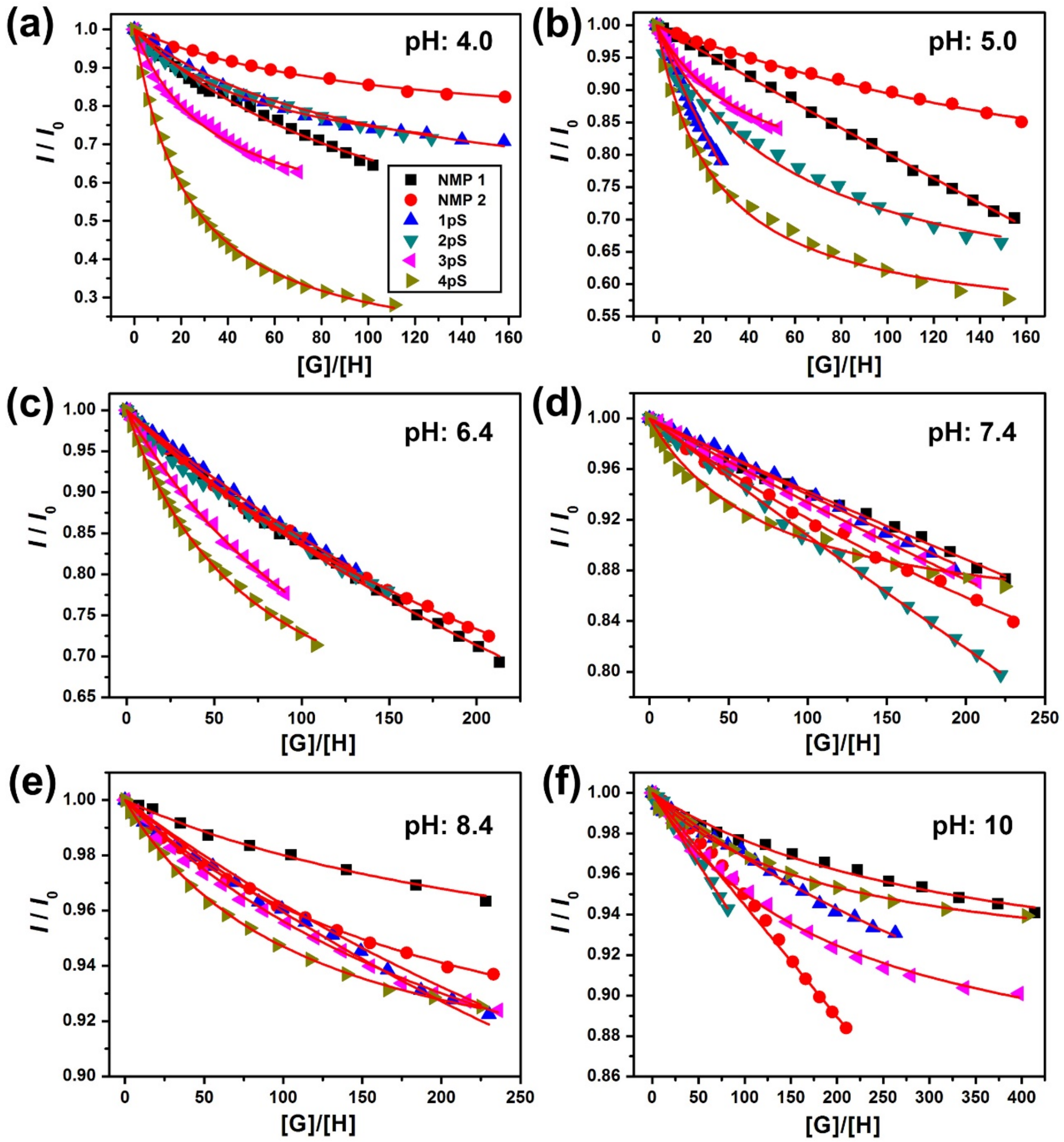


**Supplementary Figure 13 |** Fluorescent intensity changes ($I/I_0$) of various *N*-terminus fluorescein-labeled peptide hosts with the additions of different molar ratios of ATBA guest under different pH conditions (1 mmol·L$^{-1}$). (a) pH 4.0; (b) pH 5.0; (c) pH 6.4; (d) pH 7.4; (e) pH 8.4; (f) pH 10.0. The red lines shown in the figures are the nonlinear-fitted curves. [G]/[H] is an abbreviation of the molar ratio of guest to host. Peptides are discriminated by different symbols, ■: NMP 1; ●: NMP 2; ▲: serine mono-PP (1pS), ▼: di-PP (2pS), ◀: tri-PP (3pS); ▶: tetra-PP (4pS). According to the non-linear fitting calculation, $K_a$ values were obtained as listed in **Supplementary Table 4**.

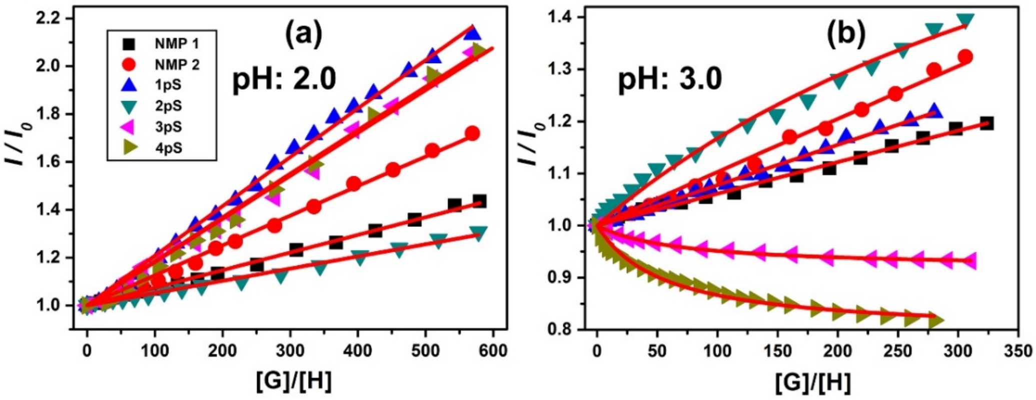


**Supplementary Figure 14 |** Fluorescent intensity changes of various *N*-terminus fluorescein-labeled peptide hosts with additions of different molar ratios of ATBA guests in buffer solutions (1 mmol·$L^{-1}$) at pH 2.0 (a) or at pH 3.0 (b). The red lines shown in the figures are nonlinear-fitted curves. [G]/[H] is an abbreviation of the molar ratio of guest to host. Peptides are discriminated by different symbols, ■: NMP 1; ●: NMP 2; ▲: serine mono-PP (1pS), ▼: di-PP (2pS), ◀: tri-PP (3pS); ▶: tetra-PP (4pS). According to the non-linear fitting calculation, $K_a$ data were obtained as listed in **Supplementary Table 4**.

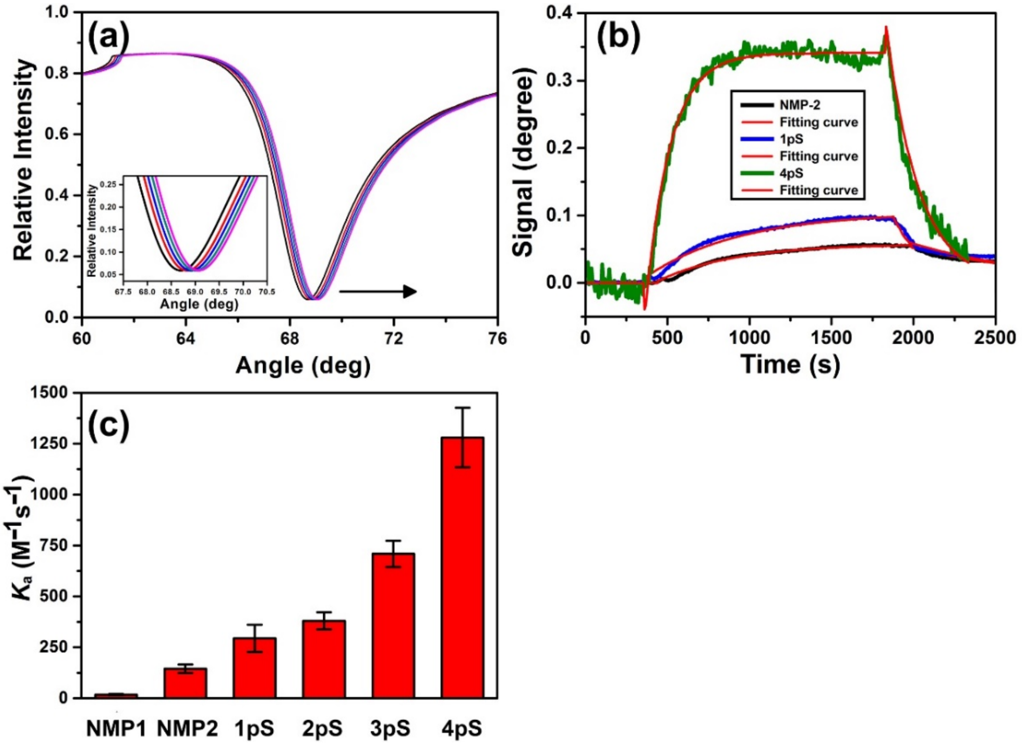


**Supplementary Figure 15 |** Association rate constant ($K_a$) measurement of peptides adsorbed on PNI-*co*-$ATBA_{0.2}$ surface, measured by Surface Plasma Resonance (SPR). (a) SPR curves of reflectance versus incident light angle (λ: 670 nm) in response to adsorption of 4pS on the copolymer–modified SPR sensor chip. (b) Time dependence of resonant angle change of copolymer–surface upon the adsorption of NMP-2 (an acidic interference peptide), 1pS or 4pS. (c) Comparison of $K_a$ of various peptides adsorbed on the copolymer surface. This data indicated that the copolymer had distinct association rate constants with these peptides.

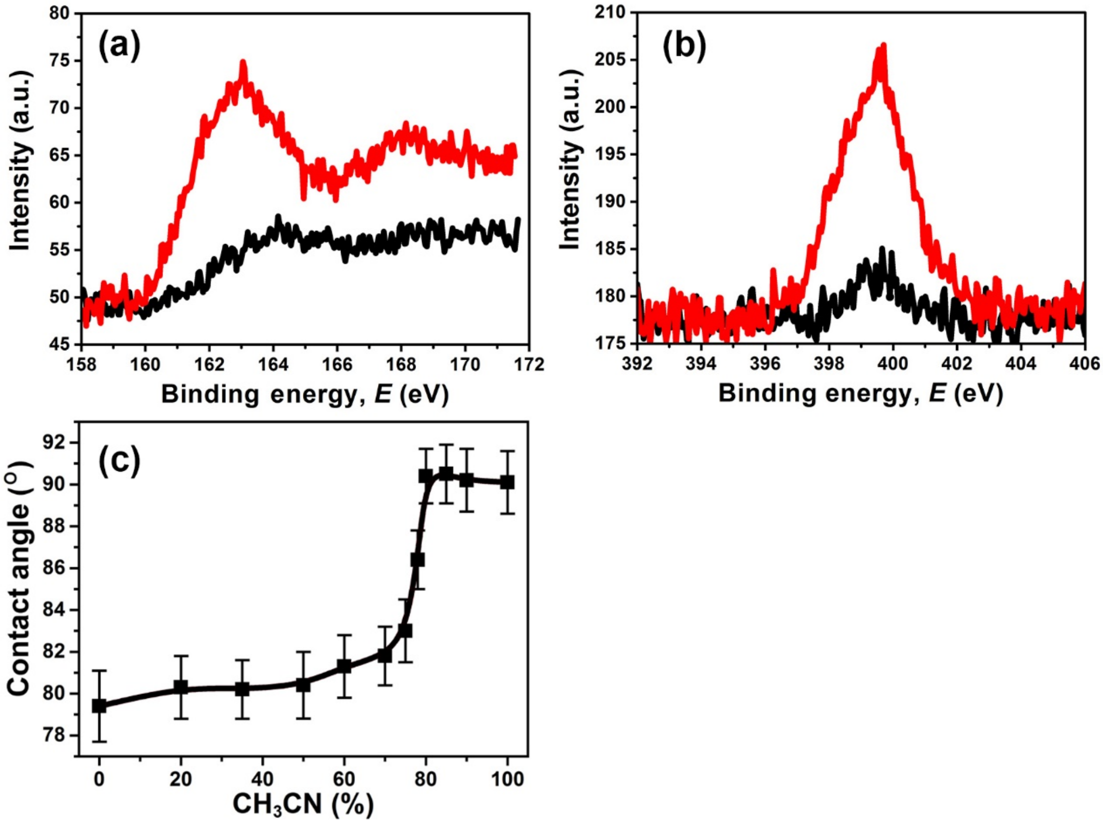


**Supplementary Figure 16 |** Sulfur (a) and nitrogen (b) element spectra of PNI-*co*-$ATBA_{0.2}$ thin film after treatment with $H_2O$ (black) or $CH_3CN$ (red), respectively, obtained by XPS measurement. (c) Acetonitrile ($CH_3CN$) content-dependent surface contact angle change of the copolymer film upon the treatment with $CH_3CN/H_2O$ mixture. Remarkable increase in the sulfur and nitrogen content, as well as the contact angle change indicated that more ATBA units exposed outside after the copolymer film was treated with $CH_3CN$, which suggested the overturn of the copolymer chains.

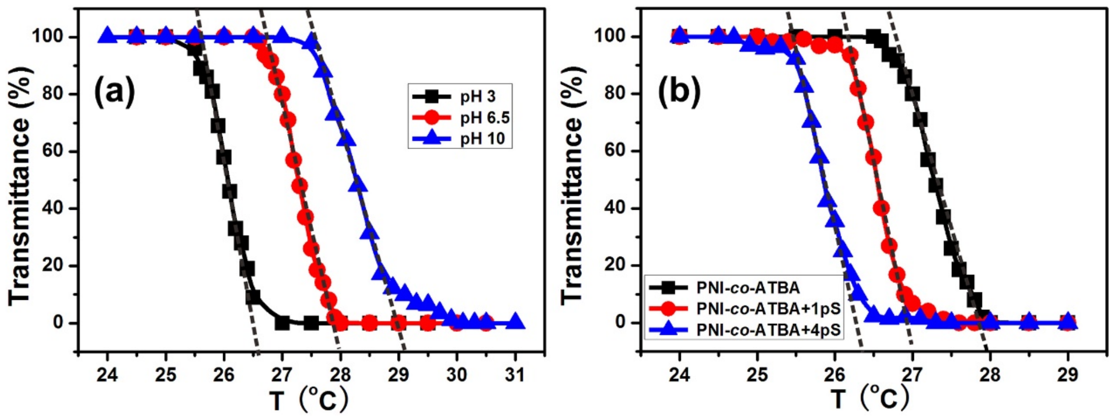


**Supplementary Figure 17 |** Transmittances of PNI-*co*-ATBA$_{0.2}$ solutions (20 mg·mL$^{-1}$) as a function of temperature. (a) Aqueous solutions with different pH values; (b) copolymer solution with addition of serine mono-PP or tetra-PP (1pS or 4pS). For (a)**,** pure water (pH~6.5) with addition of appropriate amount of hydrochloric acid (pH 3.0) or sodium hydrate (pH 10.0) was used as solution, respectively. For (b), pure water (pH~6.5) was used as solvent.

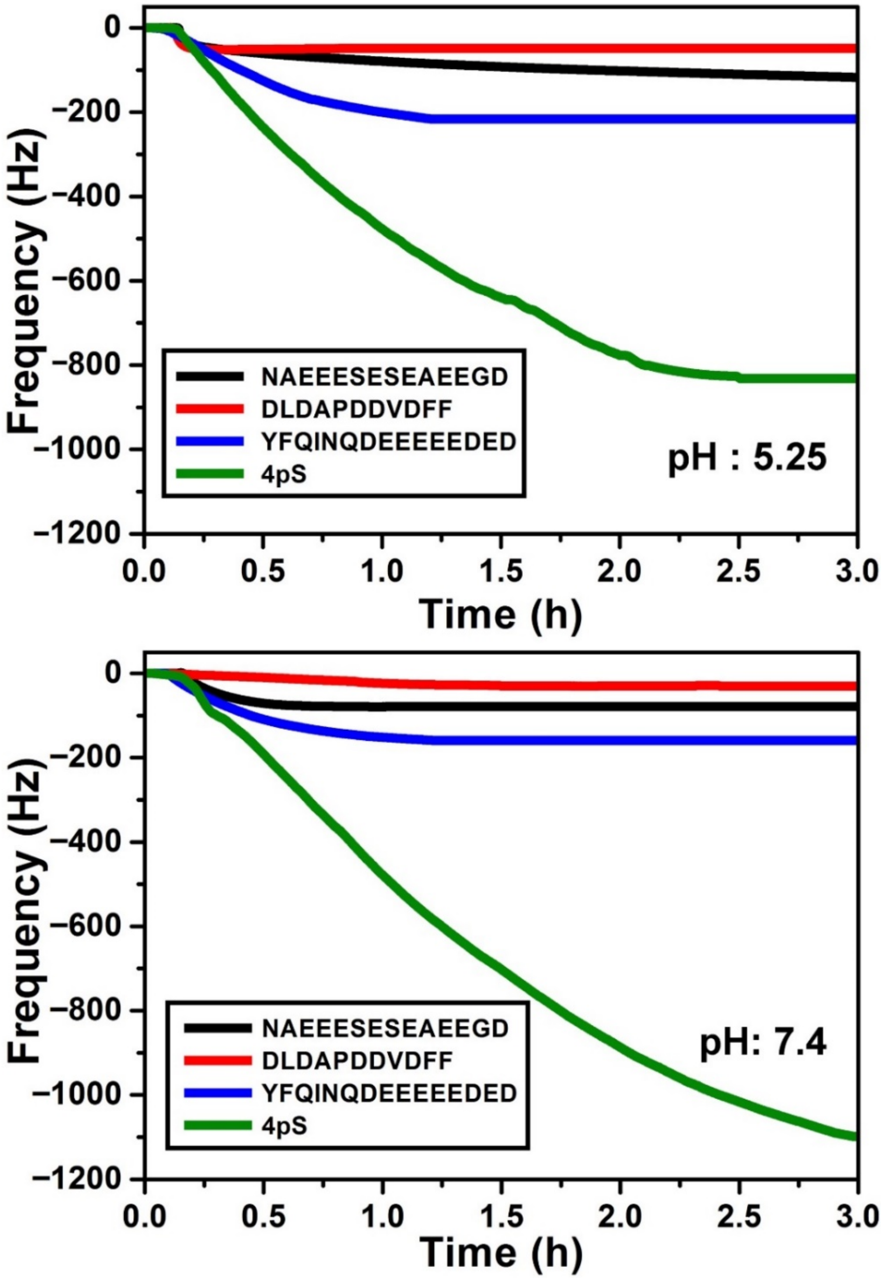


**Supplementary Figure 18 |** Time dependence of frequency change during three acidic non-modified peptides abundant with multiple Asp and/or Glu residues right next to each other, and serine tetra-PP (4pS, 75 μg·ml$^{-1}$) adsorbed on QCM resonator surfaces modified with PNI-*co*-ATBA$_{0.2}$ at different pH conditions: (a) pH: 5.25, (b) pH 7.4 in consistent solvent polarity of $CH_3CN/H_2O$ with v/v = 80:20 and temperature of 20 $^{o}$C. This data indicated the satisfactory anti-interference capacity of our material towards acidic non-modified peptides.

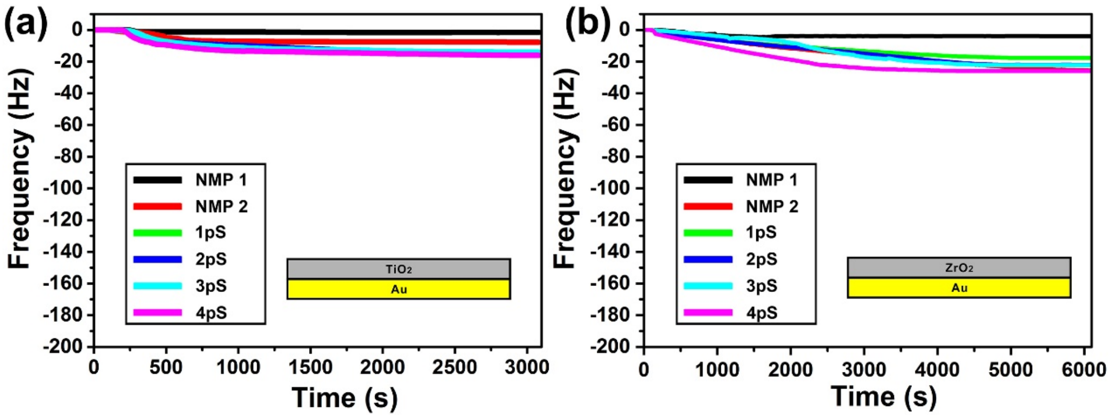


**Supplementary Figure 19 |** Time dependence of frequency change during NMPs or serine mono-, di-, tri- and tetra-PPs (1pS–4pS) adsorbed on the QCM resonator surfaces coated with a thin layer of $TiO_2$ (a) or $ZrO_2$ (b). Concentrations of the peptide solutions were 75 μg·ml$^{-1}$. $CH_3CN/H_2O$ mixture with a volume ratio of 80:20 and pH value of 7.4 was used as solvent, temperature: 20 °C. The maximum frequency change lower than 20 Hz indicated that adsorption quantities of the PPs on either $TiO_2$ or $ZrO_2$ surfaces were substantially lower than that on PNI-*co*-ATBA surfaces. Besides, the selectivity of $TiO_2$ or $ZrO_2$ towards these peptides were not satisfactory compared with that of our polymeric material.

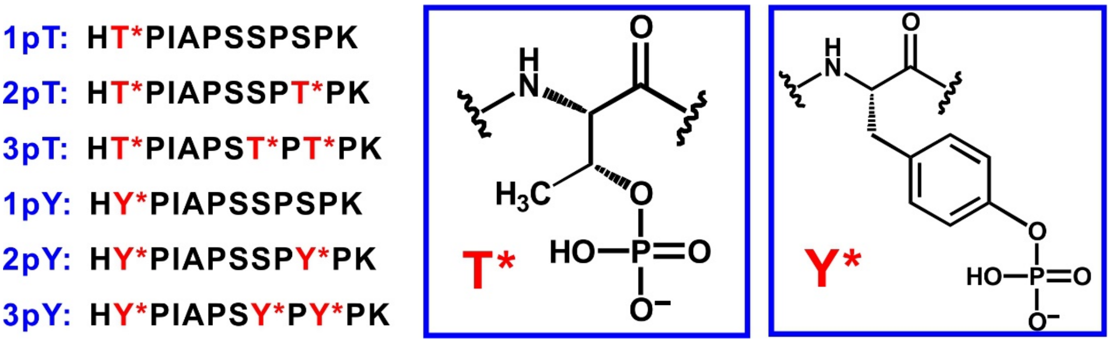


**Supplementary Figure 20 |** Amino acid sequences of threonine (1pT–3pT) or tyrosine (1pY–3pY) phosphorylated peptides (PPs) studied in this work. All these PPs were customized from China-Peptide Corp. (Shanghai, China) with high purities (> 99%), and their structures were confirmed by ESI-Q-TOF MS.

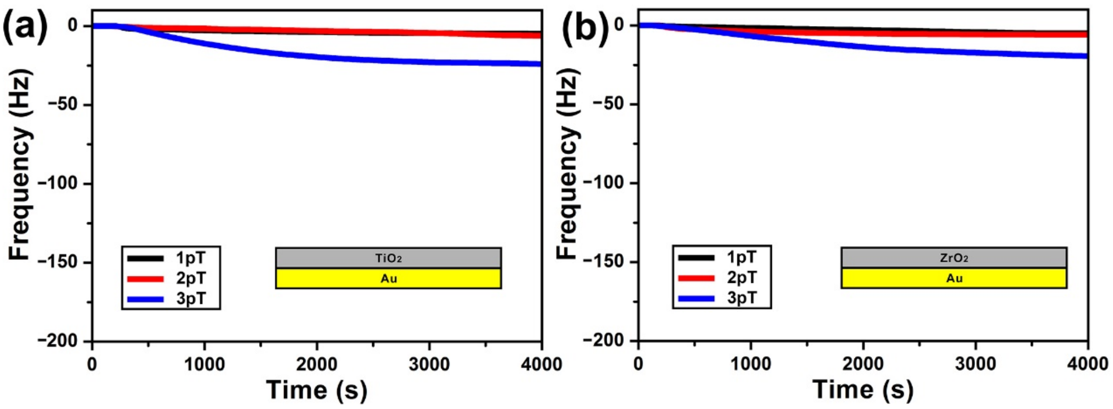


**Supplementary Figure 21 |** Time dependence of frequency change during threonine mono-, di-, and tri-PPs (1pT—3pT) adsorbed on QCM resonator surfaces coated with a thin layer of $TiO_2$ (a) or $ZrO_2$ (b). Concentrations of the peptide solutions were 75 μg·ml$^{-1}$. $CH_3CN/H_2O$ mixture with a volume ratio of 80:20 and solution pH value of 7.4 was used as solvent, temperature: 20 °C. These data indicated that adsorption quantities of 1pT—3pT on either $TiO_2$ or $ZrO_2$ surfaces were substantially lower than that observed on the PNI-*co*-$ATBA_{0.2}$ surfaces. In addition, the selectivity towards these PPs was not satisfactory compared with that of the polymeric materials. The peptide sequences of 1pT—3pT are shown in **Supplementary Figure 20**.

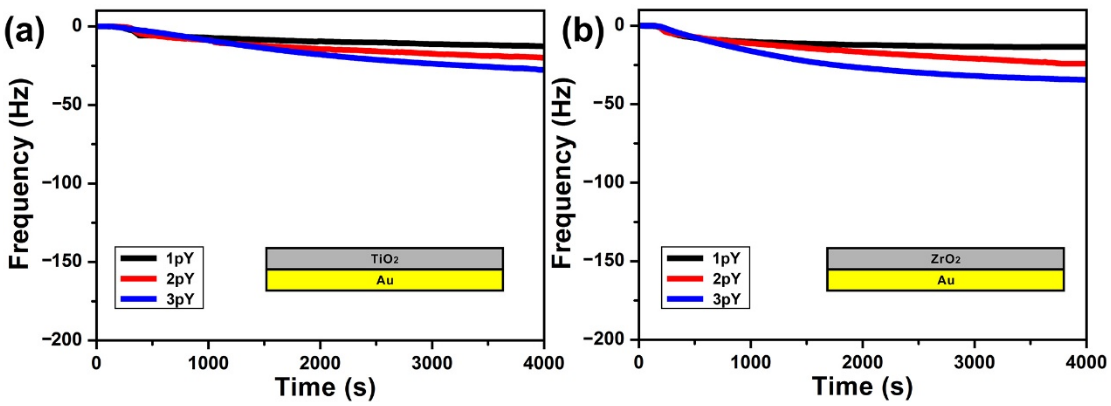


**Supplementary Figure 22 |** Time dependence of frequency change during mono-, di-, and tri-tyrosine PPs (1pY—3pY) adsorbed on QCM resonator surfaces coated with a thin layer of $TiO_2$ (a) or $ZrO_2$ (b). Concentrations of the peptide solutions were 75 μg·ml$^{-1}$. $CH_3CN/H_2O$ mixture with a volume ratio of 80:20 and solution pH value of 7.4 was used as solvent, temperature: 20 °C. The peptide sequences of 1pY—3pY are shown in **Supplementary Figure 20**.

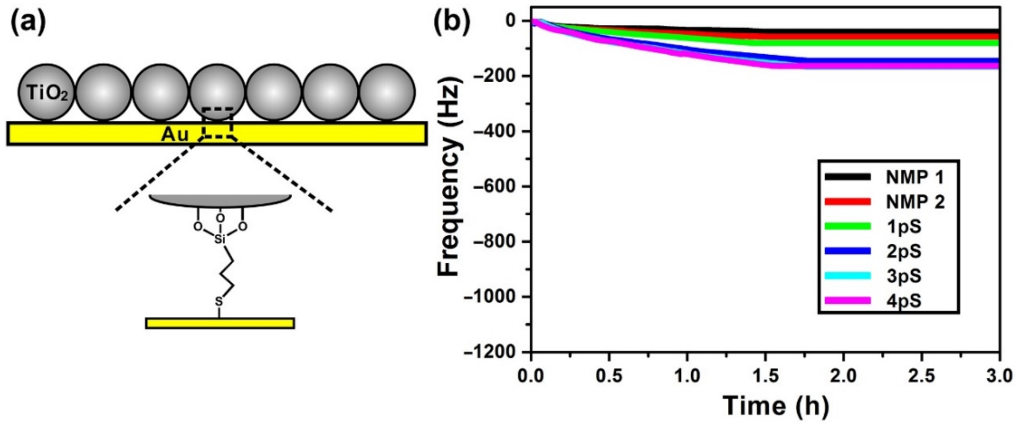


**Supplementary Figure 23 |** (a) Graphic illustration of grafting method of $TiO_2$ microspheres (product of GL Sciences, Tokyo, Japan) onto QCM resonator surface; (b) Time dependence of frequency change during various non-modified (NMP 1, 2) or serine mono-, di-, tri-, and tetra-PPs (1pS–4pS) adsorbed on the QCM resonator surfaces grafted with a thin layer of $TiO_2$ microspheres. Concentrations of the peptide solutions were 75 $\mu g \cdot ml^{-1}$. $CH_3CN/H_2O$ mixture with a volume ratio of 80:20 and solution pH value of 7.4 was used as solvent, temperature: 20 $^oC$, under this condition, the maximum adsorption of $TiO_2$ microspheres towards these PPs was observed. However, the 4pS adsorption–induced QCM frequency change was still lower than 170 Hz, which was far lower than that observed on our copolymer surface.

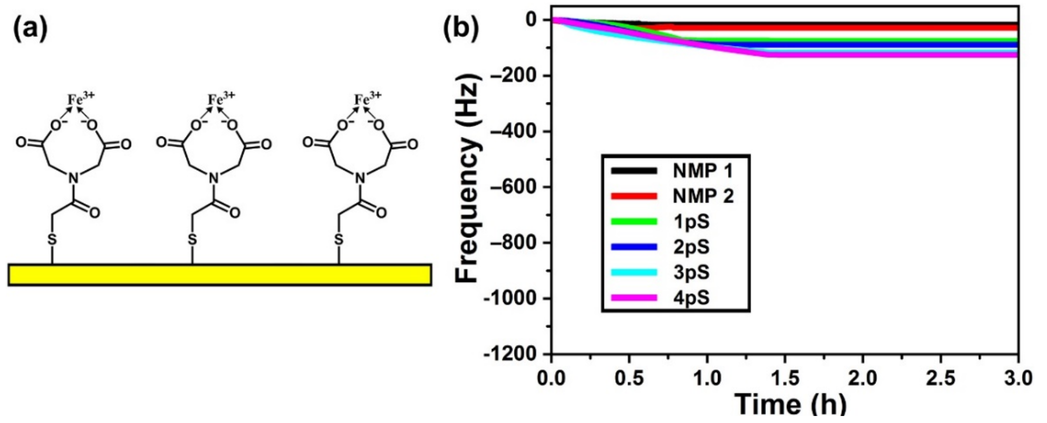


**Supplementary Figure 24 |** (a) Illustration of 2,2'-azanediyl-diacetate-$Fe^{3+}$ complex modified Au surface, which could be regarded as an analogue of IMAC material; (b) Time dependence of frequency change during various NMPs or serine mono-, di, tri-, tetra-PPs (1pS–4pS) adsorbed on the $Fe^{3+}$–complex modified QCM resonator surfaces. Concentrations of the peptide solutions were 75 $\mu g \cdot ml^{-1}$. $CH_3CN/H_2O$ mixture with a volume ratio of 80:20 was used as solvent, solution pH value: 7.4, temperature: 20 $^oC$. This experiment was designed to observe the dynamic adsorption behaviour of PPs on IMAC material. These data clearly indicated that the adsorption quantities of various PPs on $Fe^{3+}$-ligand surface were much lower than that on the PNI-*co*-ATBA$_{0.2}$ surface, while the chemoselectivity of $Fe^{3+}$-ligand modified surface towards 1pS–4pS was not notable.

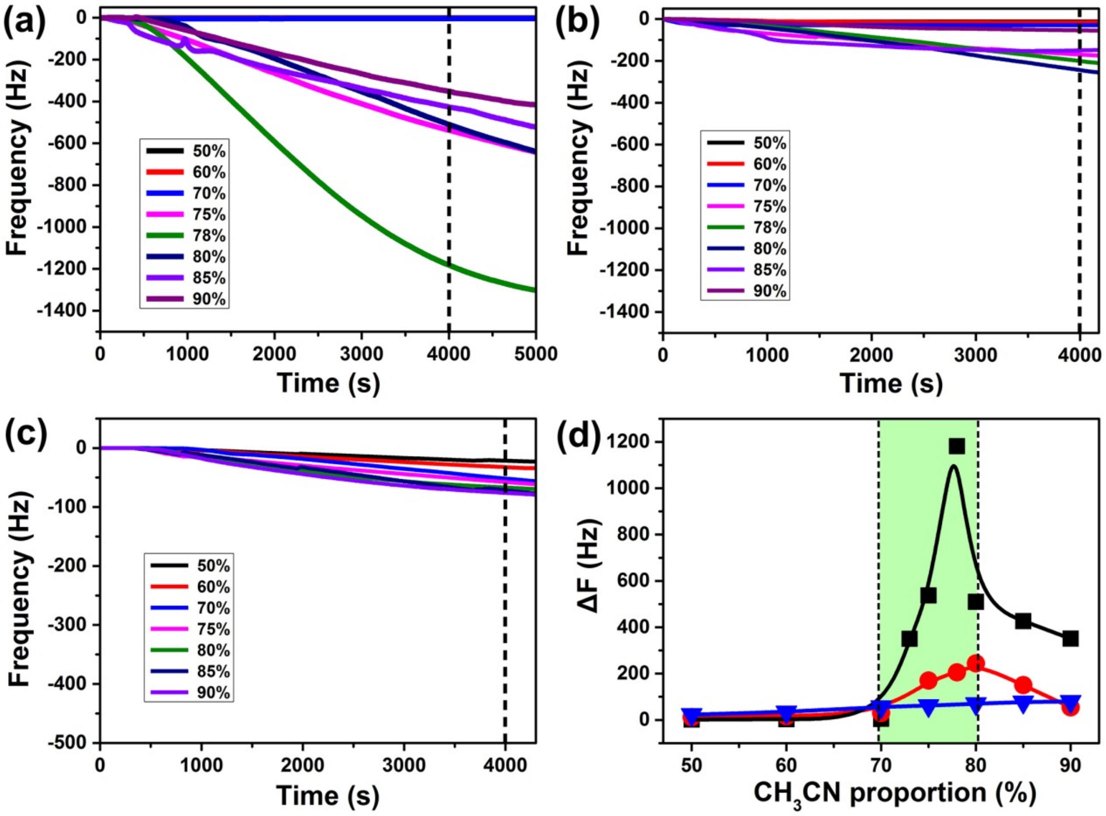


**Supplementary Figure 25 |** Time dependence of frequency change during serine tetra-PP (4pS, 75 μg·ml$^{-1}$) adsorbed on QCM resonator surfaces modified with PNI-*co*-ATBA (a), polyATBA homopolymer (b), or ATBA monolayer (c), $CH_3CN/H_2O$ mixtures with different volume ratios were used as solvent, consistent solution pH value of 7.4 and temperature of 20 $^{o}$C. According to the 4pS adsorption–induced QCM frequency changes at 4000 s, a relationship between QCM frequency change and solution polarity was built as shown in (d). Compared with polyATBA homopolymer (red circles) or ATBA monolayer (blue triangles), PNI-*co*-ATBA (black squares) displayed a dramatic adsorption conversion window when the $CH_3CN$ proportion decreased from 80% to 70%.

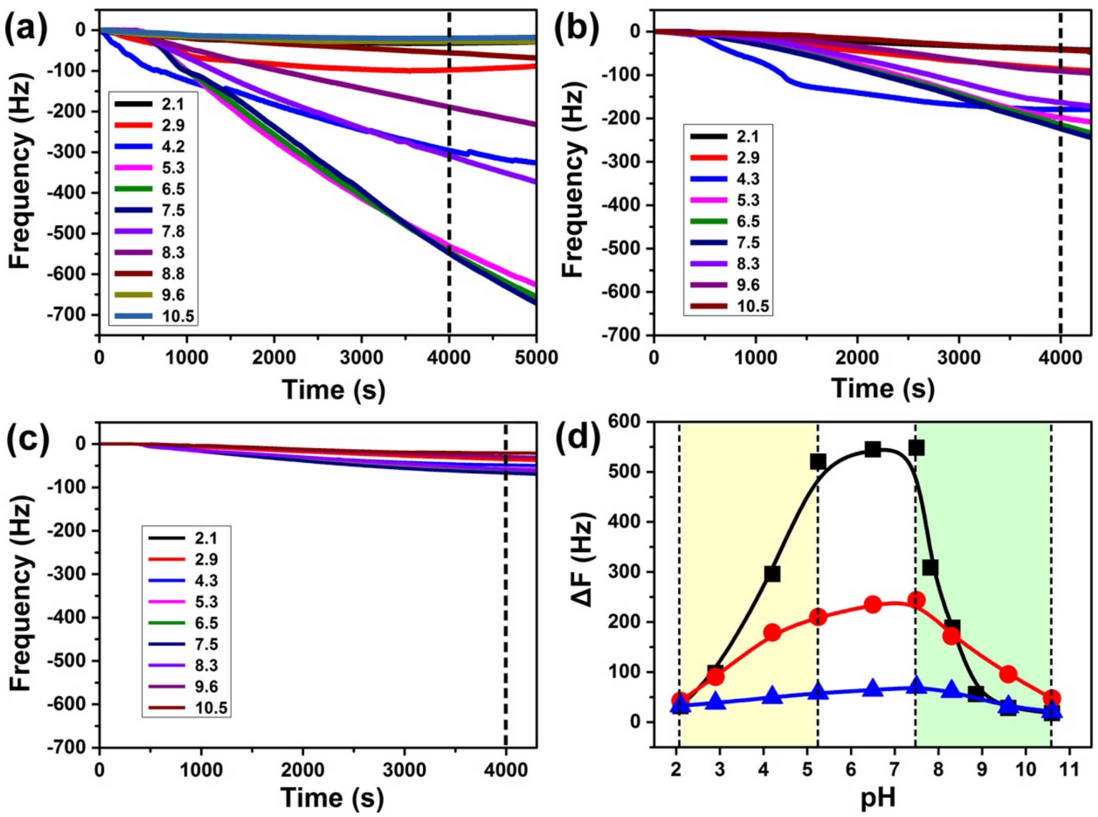


**Supplementary Figure 26 |** Time dependence of frequency change during serine tetra-PP (4pS, 75 μg·ml$^{-1}$) adsorbed on QCM resonator surfaces modified with PNI-*co*-ATBA (a), polyATBA homopolymer (b), or ATBA monolayer (c). $CH_3CN/H_2O$ mixtures with different pH values were used as solvent, consistent solvent polarity of $CH_3CN/H_2O$ with v/v = 80:20 and temperature of 20 $^{o}$C. According to the 4pS adsorption-induced QCM frequency change at 4000 s, a relationship (d) between QCM frequency change and solution pH value was built. In (d), dramatic adsorption conversion windows were observed in pH range of 2—5.2 and 7.4—9 for PNI-*co*-ATBA (black squares), similar conversion window was not observed for polyATBA (red circles) or ATBA monolayer (blue triangles) surface.

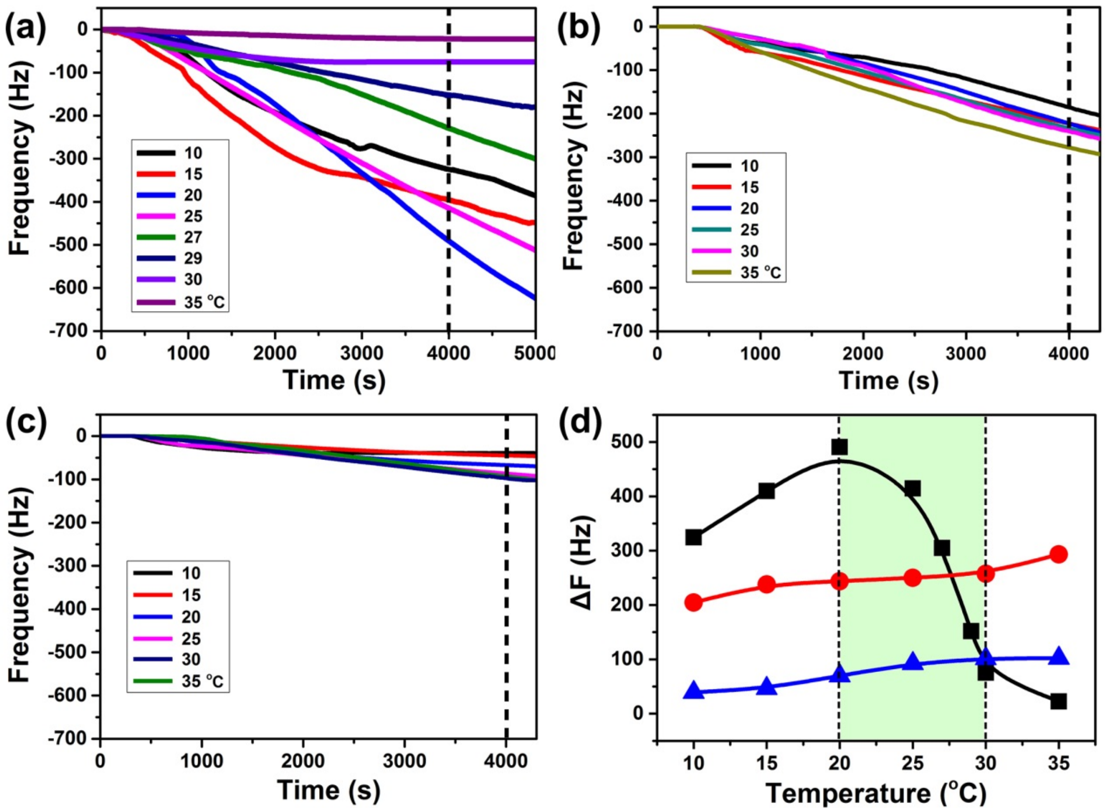


**Supplementary Figure 27 |** Time dependence of frequency change during serine tetra-PP (4pS, 75 μg·ml$^{-1}$) adsorbed on QCM resonator surfaces modified with PNI-*co*-ATBA (a), polyATBA homopolymer (b), or ATBA monolayer (c) at different temperature from 10 to 35 $^{o}$C. Consistent $CH_3CN/H_2O$ mixture (volume ratio of 80:20, Tris-HCl solution, pH 7.4) worked as solvent. These data clearly indicated that PNI-*co*-ATBA (black squares) exhibited excellent thermo-responsiveness and distinct adsorption capacities towards 4pS at different temperature (d). Control experiments using polyATBA or ATBA monolayer further revealed that temperature-modulated PP adsorption behaviours of PNI-*co*-ATBA originated from the "*coil*-to-*globule*" transition of flexible PNIPAAm chain, because the adsorption behaviours of 4pS on polyATBA (red circles) and ATBA (blue triangles) surface could not be modulated by temperature.

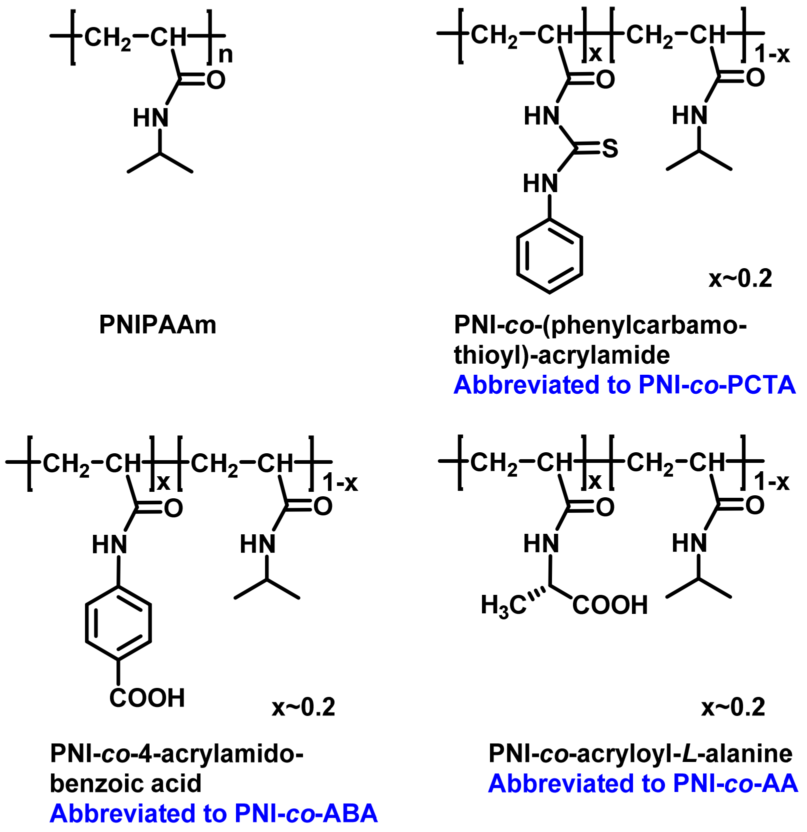


**Supplementary Figure 28 |** Chemical structures of four reference polymers, namely PNIPAAm, PNI-*co*-PCTA$_{0.2}$, PNI-*co*-ABA$_{0.2}$, PNI-*co*-AA$_{0.2}$, which were used in QCM adsorption experiment in order to validate the rationality of our copolymer design.

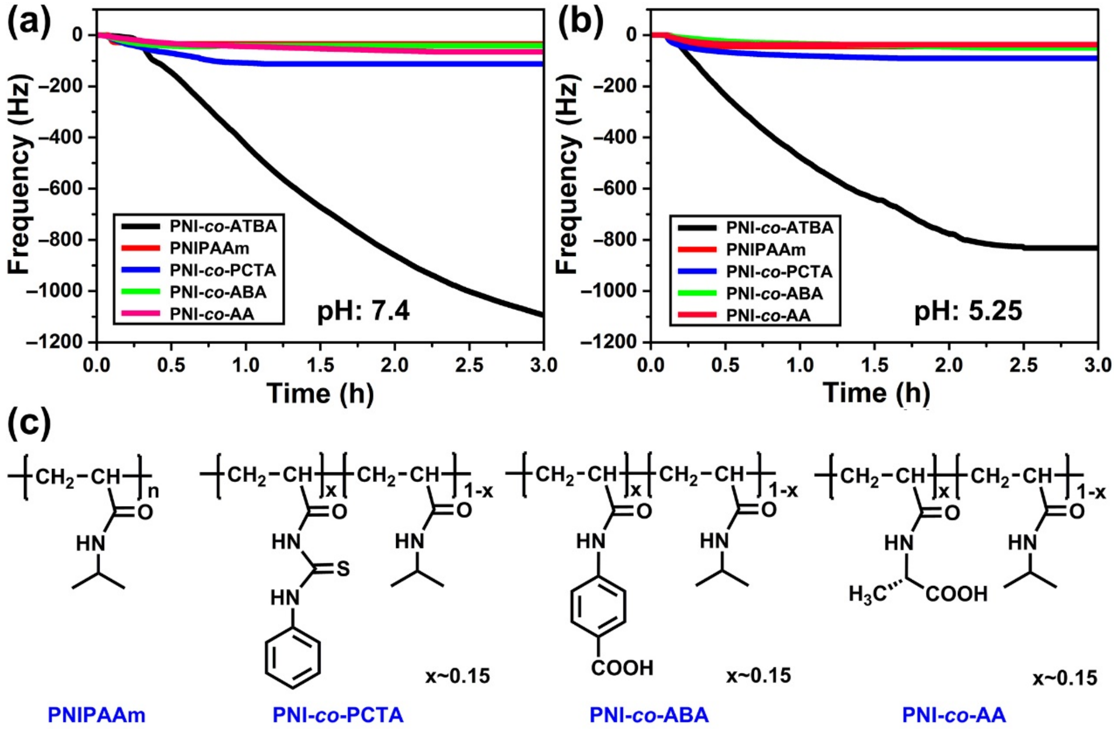


**Supplementary Figure 29 |** (a, b) Time dependence of frequency change during serine tetra-PP (4pS) adsorbed on QCM resonator surfaces modified with various reference polymers (i.e., PNIPAAm, PNI-*co*-PCTA, PNI-*co*-ABA, and PNI-*co*-AA). Consistent solvent polarity of $CH_3CN/H_2O$ v/v: 4:1 and temperature at 20 °C, but different solution pH of 7.4 (a) and 5.25 (b) were used. (c) Chemical structures of the reference polymers.

Compared with the excellent performance of PNI-*co*-$ATBA_{0.2}$, the adsorption quantities of 4pS on these reference polymer thin film were quite low ($\Delta F_{max}$ < 115 Hz). The absence of ATBA or carboxylic acid, thiourea or phenyl group in ATBA, would induce substantial decreases in the 4pS adsorption quantities on PNIPAAm, PNI-*co*-PCTA, PNI-*co*-ABA, PNI-*co*-AA surface, respectively. The control experiments illustrated the rationality of the design of the ATBA functional unit. We presumed that both thiourea and carboxylic acid functioned as binding groups towards phosphates in 4pS, while these binding groups were closely interconnected by a rigid phenyl group, which further strengthened the hydrogen bonding interactions with phosphates. All these functional groups in ATBA constituted a synergetic and highly efficient system to facilitate a strong combination with 4pS.

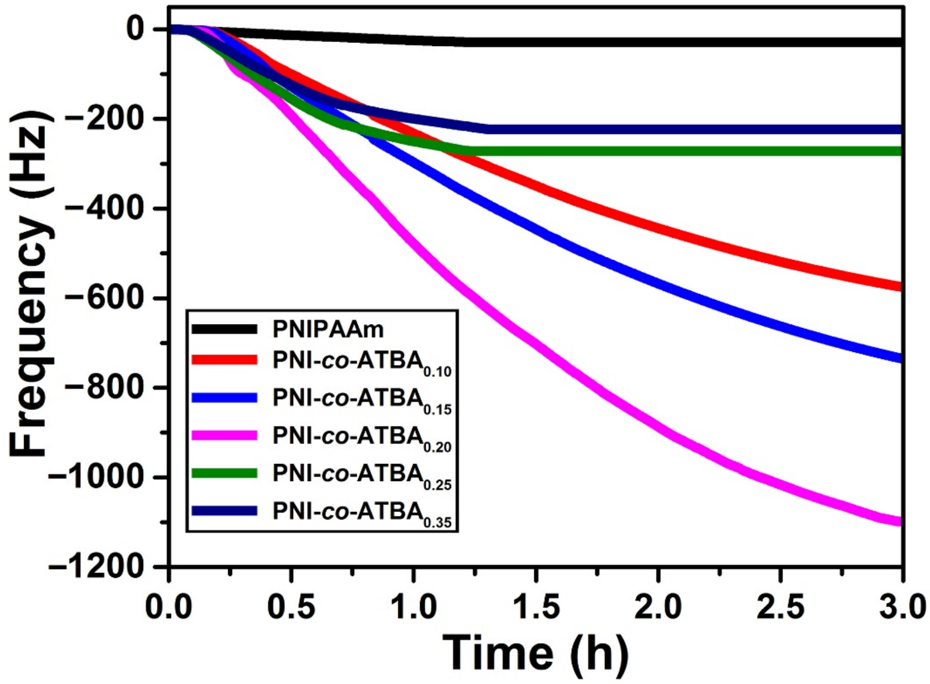


**Supplementary Figure 30 |** Time dependence of frequency change during serine tetra-PP (4pS) adsorbed on QCM resonator surfaces modified with PNI-*co*-ATBA$_x$ the ATBA ratio (x) ranges from.0 to 0.35. Consistent solvent polarity of $CH_3CN/H_2O$ (v/v: 4:1), pH 7.4 and temperature at 20 °C.

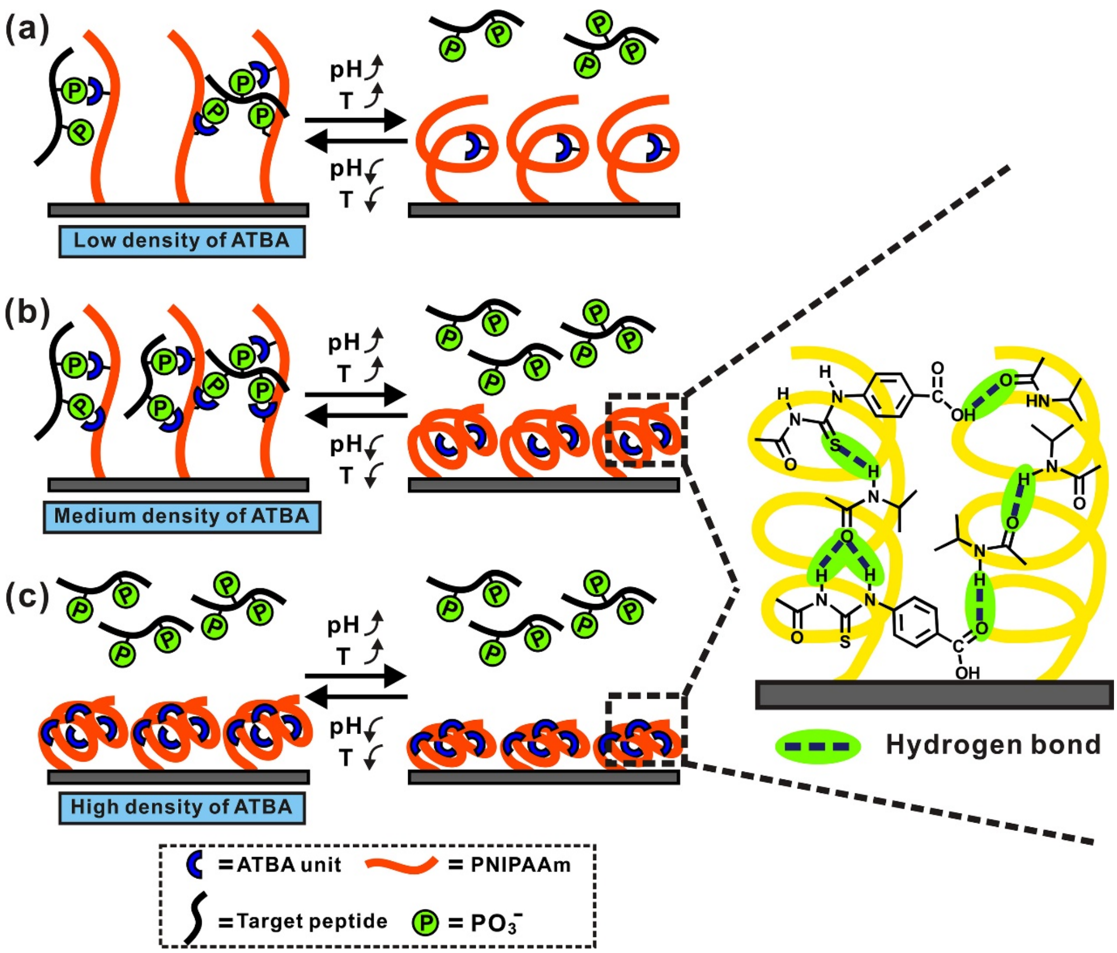


**Supplementary Figure 31 |** Graphic illustration of the role of ATBA content in the adsorption performance of the copolymer film towards MPPs. (a) For the lower ATBA content (i.e., 10% or 15%), insufficient ATBA binding units are grafted in the polymer chains, which substantially reduce the adsorption performance of the copolymer film. (c) By contrast, for the higher ATBA content (i.e., 25% or 35%), due to intensive intermolecular hydrogen bonding interactions among ATBA and NIPPAm units, a contracted and compact polymer film was constructed. Under this condition, only small amounts of ATBA units remain exposed and can bind to MPPs, resulting a sharp decrease in adsorption capacity towards MPPs. (b) The optimal ATBA content in the copolymer was determined to be 20%, sufficient ATBA binding groups and favorable polymer conformational transition would largely promote the MPPs adsorption on the copolymer film.

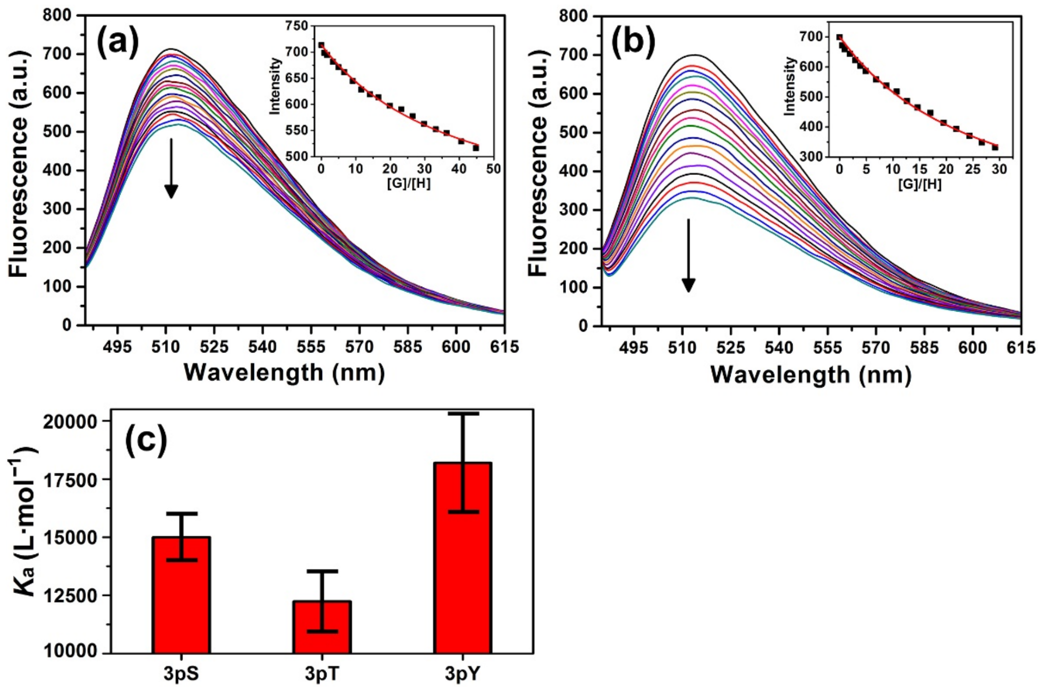


**Supplementary Figure 32 |** (a, b) Typical fluorescence spectra of *N*-terminus fluorescein-labeled threonine tri-PP (a, 3pT) or tyrosine tri-PP (b, 3pY) (concentration: $2.0 \times 10^{-6}$ mol·L$^{-1}$) host upon additions of different equivalents of ATBA monomer guests in formate-buffer solution (pH 4.0, 1 mmol·L$^{-1}$) at 20 °C. The insets show the fluorescent intensity changes of 3pT or 3pY host upon the additions of ATBA guest. The red lines are nonlinear-fitted curves. [G]/[H] is an abbreviation of molar ratio of guest to host. The $K_a$ of 3pT or 3pY with ATBA was 12237 ± 1293 or 18195 ± 2120 L·mol$^{-1}$, respectively. (c) Comparison of $K_a$ of ATBA with 3pS, 3pT and 3pY in formate-buffer solution (pH 4.0, 1 mmol·L$^{-1}$) at 20 °C.

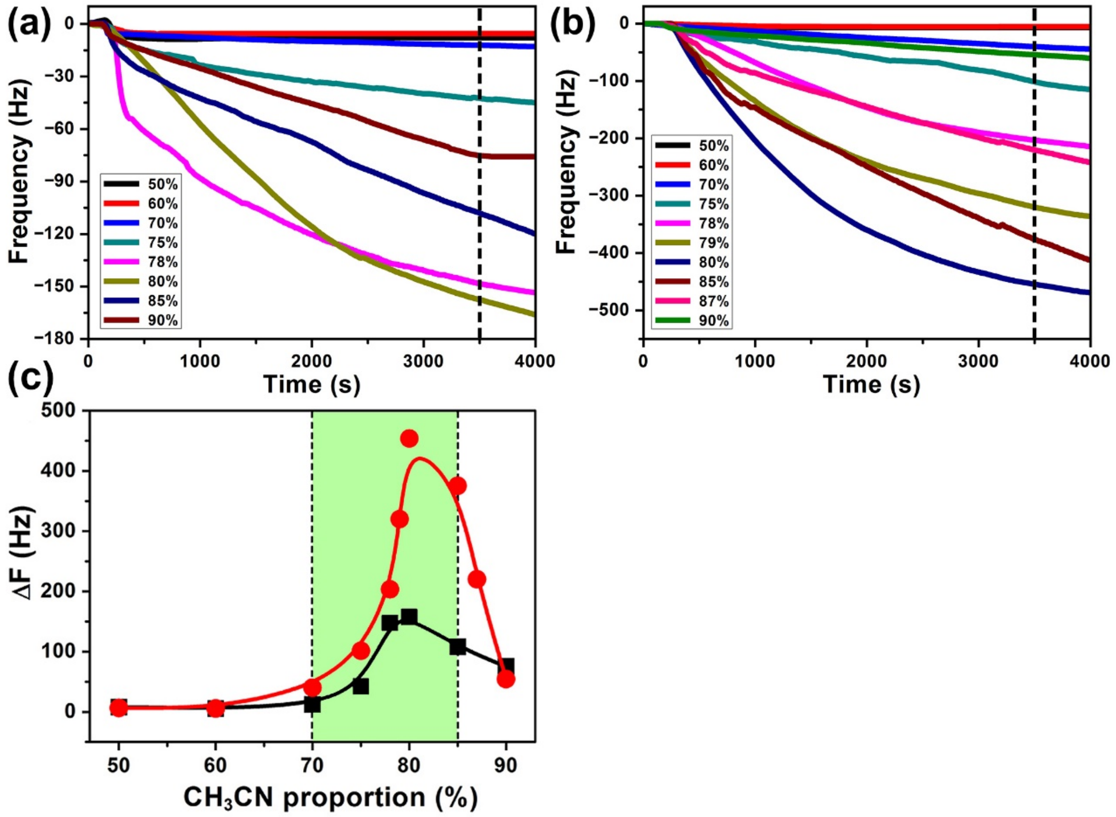


**Supplementary Figure 33 |** Time dependence of frequency change during threonine tri- (3pT) (a) or tyrosine tri-PP (3pY) (b) adsorbed on QCM resonator surfaces modified with PNI-*co*-ATBA$_{0.2}$. Concentrations of the PP solutions were 75 μg·ml$^{-1}$, and $CH_3CN/H_2O$ mixtures with different volume ratios were used as solvent, solution pH value: 7.4 and temperature: 20 °C. (c) Solvent polarity-dependent QCM frequency changes in response to the adsorption of 3pT (black squares) or 3pY (red circles) on the PNI-*co*-ATBA$_{0.2}$ surface. Two solvent polarity-mediated adsorption conversion windows for 3pT or 3pY were observed as indicated by a green layer. 3pT sequence: H(p-T)PIAPS(p-T)P(p-T)PK; 3pY sequence: H(p-Y)PIAPS(p-Y)P(p-Y)PK.

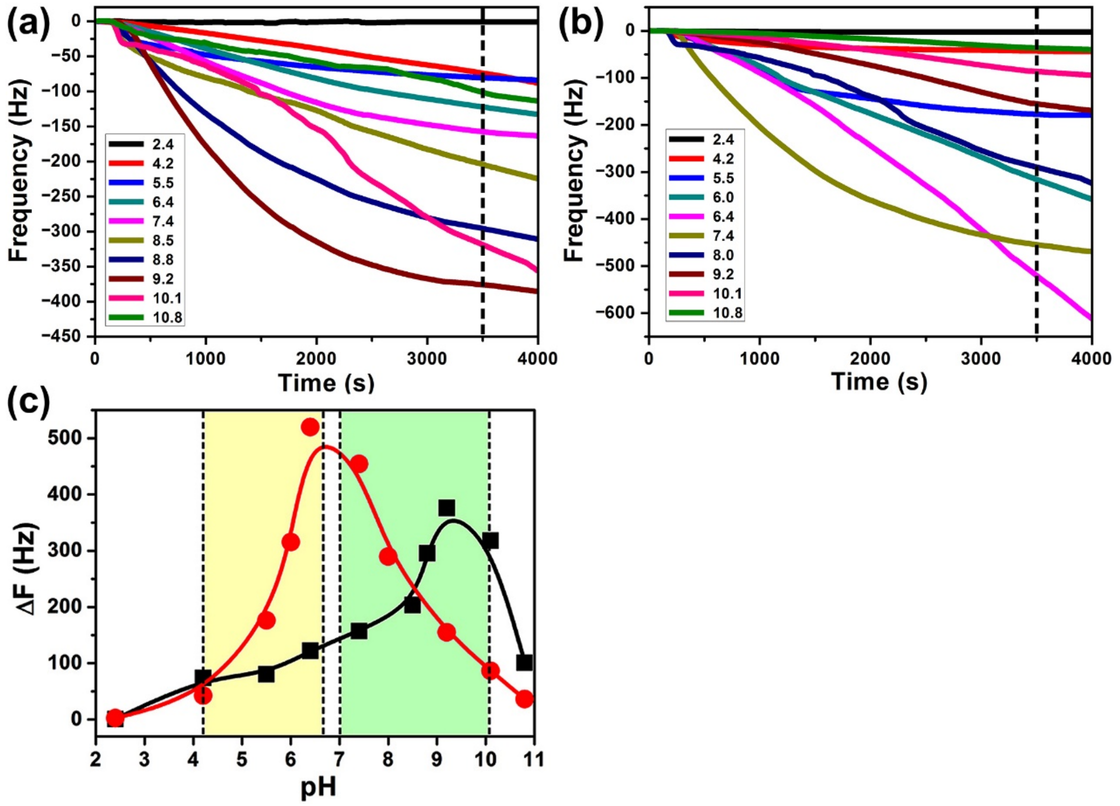


**Supplementary Figure 34 |** Time dependence of frequency change during threonine tri- (3pT) (a) or tyrosine tri-PP (3pY) (b) adsorbed on QCM resonator surfaces modified with PNI-*co*-ATBA$_{0.2}$. Concentrations of the PP solutions were 75 μg·ml$^{-1}$, and $CH_3CN/H_2O$ mixtures with different solution pH were used as solvent, solvent polarity: $CH_3CN/H_2O$: v/v = 4:1, temperature: 20 °C. (c) Solution pH–dependent QCM resonator frequency change in response to the adsorption of 3pT (black squares) or 3pY (red circles) on the PNI-*co*-ATBA$_{0.2}$ surface. Two pH-mediated adsorption conversion windows for 3pY were observed. The former one (indicated by yellow layer) could be attributed to the pH-mediated binding affinity change of ATBA with 3pY, strong acidity (pH lower than 3) would destroy the hydrogen bonding network of the copolymer and result in the sharp decrease in 3pY adsorption quantity. The second adsorption conversion window (indicated by green layer) corresponds to the conformation transition of the polymeric chain in response to the pH change, the adsorbed 3pY would be released from the contracted polymeric network. 3pT sequence: H(p-T)PIAPS(p-T)P(p-T)PK; 3pY sequence: H(p-Y)PIAPS(p-Y)P(p-Y)PK.

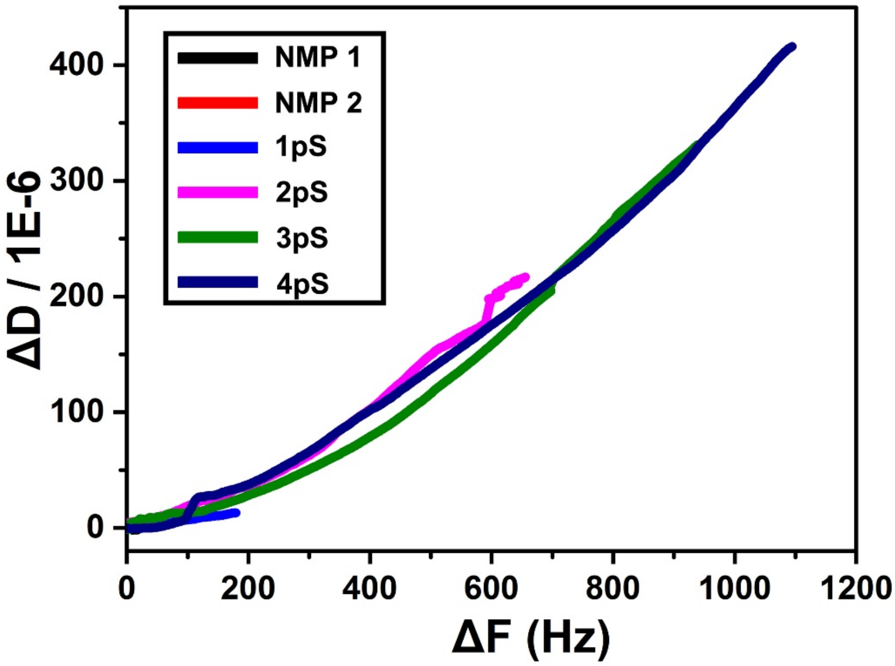


**Supplementary Figure 35 |** Viscoelasticity property study of copolymer film monitored by QCM-D. Relationship between shifts in dissipation (ΔD) and frequency (ΔF) for adsorption of NMPs and serine PPs (i.e. 1pS–4pS) on QCM resonator modified with PNI-*co*-$ATBA_{0.2}$. Concentrations of the peptide solutions were 75 μg·$ml^{-1}$, and $CH_3CN/H_2O$ mixture with a volume ratio of 80:20 was used as solvent, solution pH value of 7.4 and at 20 °C.

Energy dissipation (D) during the oscillation of resonator is an indication of the rigidity of the adsorbed layer.[7] The increase of dissipation indicated that the adsorbed layer became less rigid. Thus, we presumed that the copolymer chains became more relaxed and expanded in response to the MPP adsorption. This presumption was further proven by the relationships between ΔD and ΔF corresponding to the adsorption processes (**Supplementary Figure 35**). The ΔD/ΔF curve indicates the formation of a viscoelastic and hydrated layer, a positive shift in the slope denotes the formation of a more viscoelastic and less rigid layer.[7] Based on this knowledge, 4pS-adsorption triggered a rapid and obvious expansion of the copolymer chains, accompanying with dramatic change in viscoelasticity and Frigidity of the copolymer film. In comparison, no evidential change of the copolymer film was observed in response to the NMP 1, 2 or 1pS adsorption. This result was consistent with the AFM data shown in **Supplementary Figure 36**, in which the film thickness increased by 74% after being immersed in the 4pS solution.

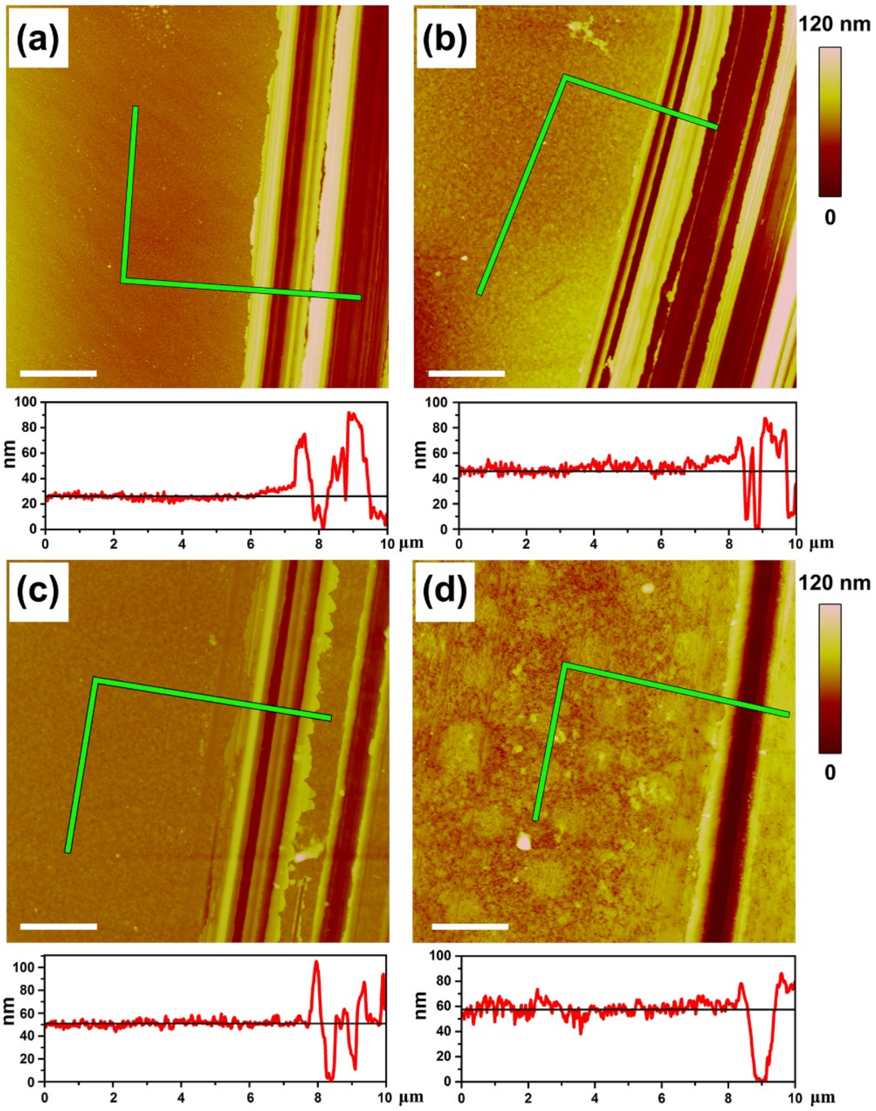


**Supplementary Figure 36 |** Typical atomic force microscopy (AFM) images of QCM resonator (a), PNI-*co*-ATBA$_{0.2}$-modified QCM resonator before (b) and after being immersed in a solution of serine mono-PP (c, 1pS) or tetra-PP (d, 4pS) (Concentrations: 75 μg·ml$^{-1}$ in Tris-HCl buffer solution, pH 7.4) for 1 h at 20 °C, and the corresponding section profiles along the green lines in the AFM images. Scale bars: 2 μm. The QCM-resonator was coated with a thin layer of gold with an average thickness of 26 ± 1 nm (a). After immobilizing PNI-*co*-ATBA$_{0.2}$ onto the QCM resonator, the cumulative film thickness increased to 45 ± 2.5 nm (b), which corresponded to a copolymer film thickness of approximately 19 nm. When this film was immersed in a 1pS solution for 1 h, no evidential change in surface morphology was detected except a slight increase in the film thickness from 19 nm to 24 nm after subtracting the gold-layer thickness (c). By comparison, the cumulative thickness remarkably increased to 59 ± 2 nm (d) when the film was treated with 4pS solution under the same condition. This could be attributed to the evidential swelling behavior of the copolymer film, accompanying with an evidential increase in the copolymer film thickness from 19 nm to 33 nm.

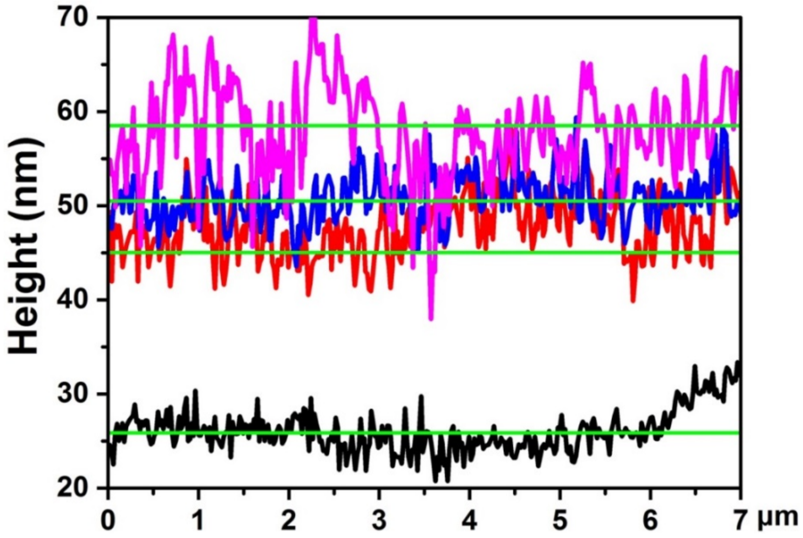


**Supplementary Figure 37 |** Comparison of film thickness of blank Au layer (black), PNI-*co*-ATBA$_{0.2}$ modified Au layer before (red) and after being treated with a serine mono-PP (blue, 1pS), or tetra-PP (purple, 4pS) solution for 1 h at 20 °C. Concentrations: 75 μg·ml$^{-1}$ in Tris-buffer, pH 7.4. The section profiles were obtained from the AFM images shown in **Supplementary Figure 36**.

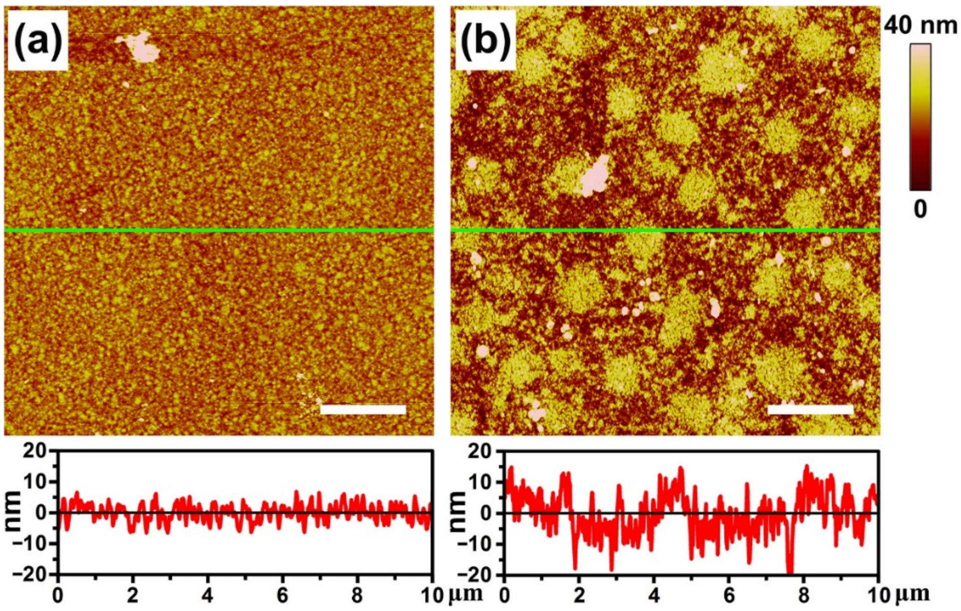


**Supplementary Figure 38 |** Typical atomic force microscopy (AFM) images of PNI-*co*-ATBA$_{0.2}$ film before (a) and after (b) treatment with a serine tetra-PP (4pS) solution (Concentration: 37.5 μg·ml$^{-1}$ in $CH_3CN/H_2O$ (v/v: 80:20) Tris-HCl buffer solution at pH 7.4) for 1 h at 20 °C, as well as the corresponding section profiles along the green lines in the AFM images. Scale bars: 2 μm. This data indicated that remarkable morphological change was also observed when the polymeric film was immersed in a $CH_3CN/H_2O$ mixture containing 4pS, which was consistent with the phenomena observed in pure water, as shown in **Figure 3a** and **3b** in the manuscript.

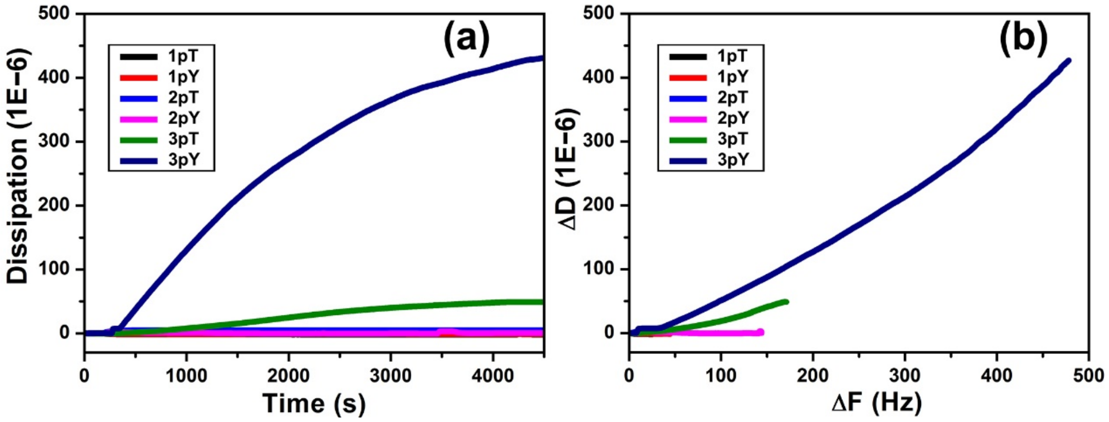


**Supplementary Figure 39 |** (a) Time dependence of dissipation changes during threonine (1pT—3pT) or tyrosine (1pY—3pY) mono-, di- or tri-PPs adsorbed on QCM resonator surfaces modified with PNI-*co*-ATBA$_{0.2}$. Concentrations of PPs: 75 μg·ml$^{-1}$, $CH_3CN/H_2O$ mixture with a volume ratio of 80:20 was used as solvent, solution pH value of 7.4 and temperature at 20 °C. (b) Relationship between shifts in dissipation (ΔD) and frequency (ΔF) for the adsorption of various PPs on the PNI-*co*-ATBA$_{0.2}$ modified QCM resonator. These data indicated that the copolymer chains occurred evidential expansion in response to the 3pY adsorption.

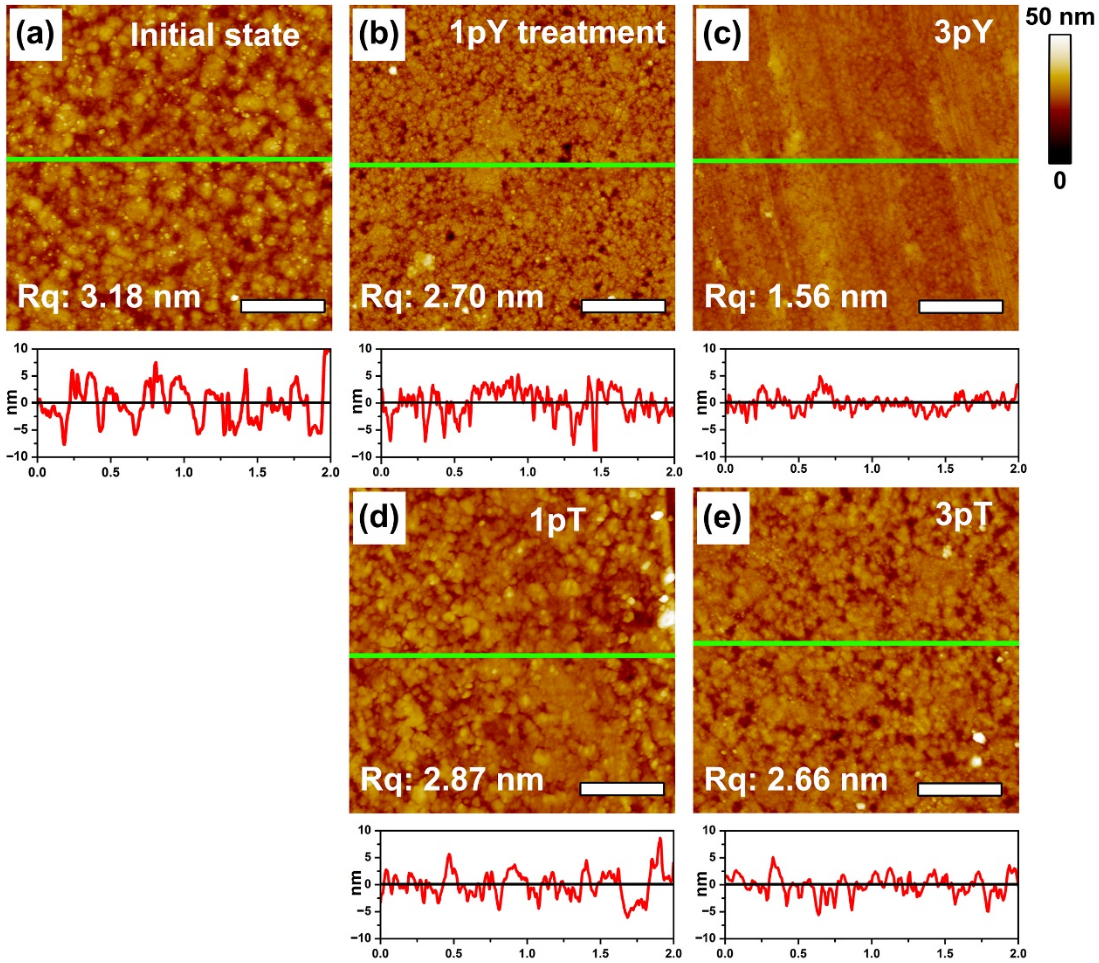


**Supplementary Figure 40 |** AFM images of PNI-*co*-ATBA$_{0.2}$ thin films before (a) and after being treated with tyrosine (b, c) or threonine (d, e) mono- (b, d) or tri- (c, e) PP solution (Concentrations of PPs: 75 μg·ml$^{-1}$ in Tris-HCl buffer, pH: 7.4) for 1 h at 20 °C, and the corresponding section profiles along the green lines in the AFM images. Scale bars: 500 nm. Remarkable surface morphology change and the decrease in surface roughness (Rq values) indicated that the adsorption of 3pY or 3pT induced remarkable expansion of the polymeric films, resulting in more smooth surfaces with less roughness. Rq is an abbreviation of root mean square roughness.

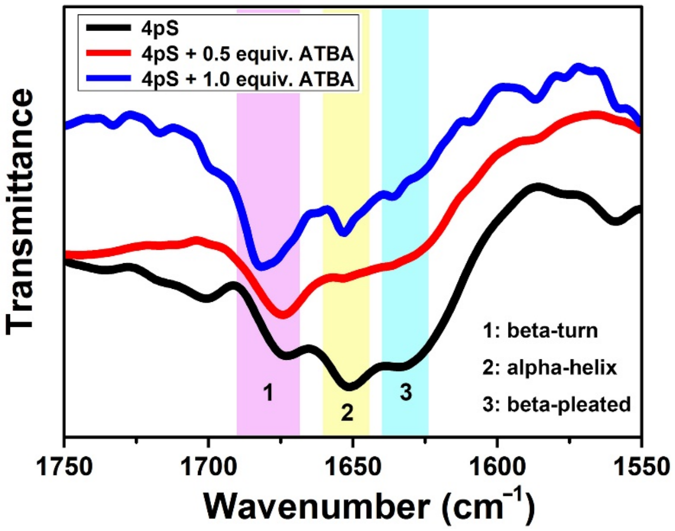


**Supplementary Figure 41 |** Representative FT–IR spectra of serine tetra-PP (4pS, 2 × $10^{-4}$ mol·$L^{-1}$) interacted with different amounts of ATBA in $D_2O$ at 20 °C. Color ribbons illustrate three characteristic amide I peaks of 4pS, which correspond to the secondary structures of peptide, 1: β-turn, 2: α-helix, 3: β-pleated sheet. This data suggested the evidentially conformational change of 4pS interacted with ATBA. In which, the proportions of α-helix and β-pleated sheet structures of 4pS decreased gradually; by comparison, the peak of β-turns structure increased remarkably, accompanying with an obvious red-shift from 1674 to 1682 $cm^{-1}$.

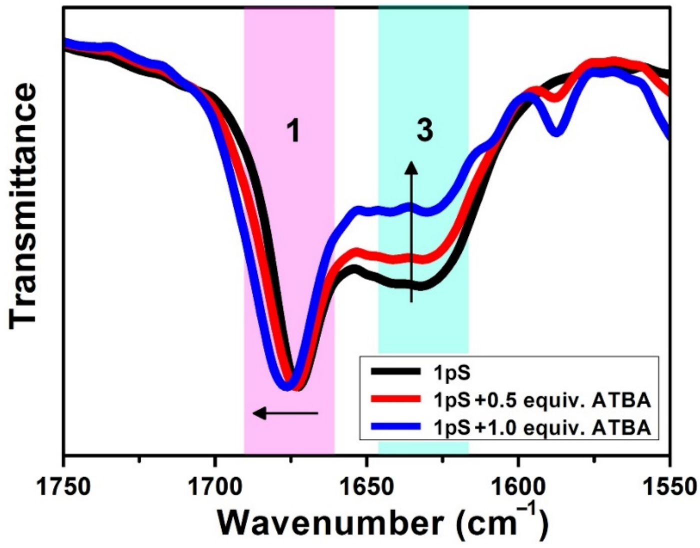


**Supplementary Figure 42 |** Representative FT–IR spectra of serine mono-PP (1pS, 2 × $10^{-4}$ mol·$L^{-1}$) interacted with different amounts of ATBA in $D_2O$ at 20 °C. Color ribbons illustrate two characteristic amide I peaks of 1pS, which correspond to the secondary structure of peptide, 1: β-turns and 3: β-pleated sheet. This data suggested that the proportion of β-pleated sheet structure of 1pS decreased gradually; while the peak of β-turn structure displayed a slight red-shift from 1672 to 1677 $cm^{-1}$. Compared with the evidential change of 4pS, the conformational change of 1pS was substantially weaker when it interacted with ATBA.

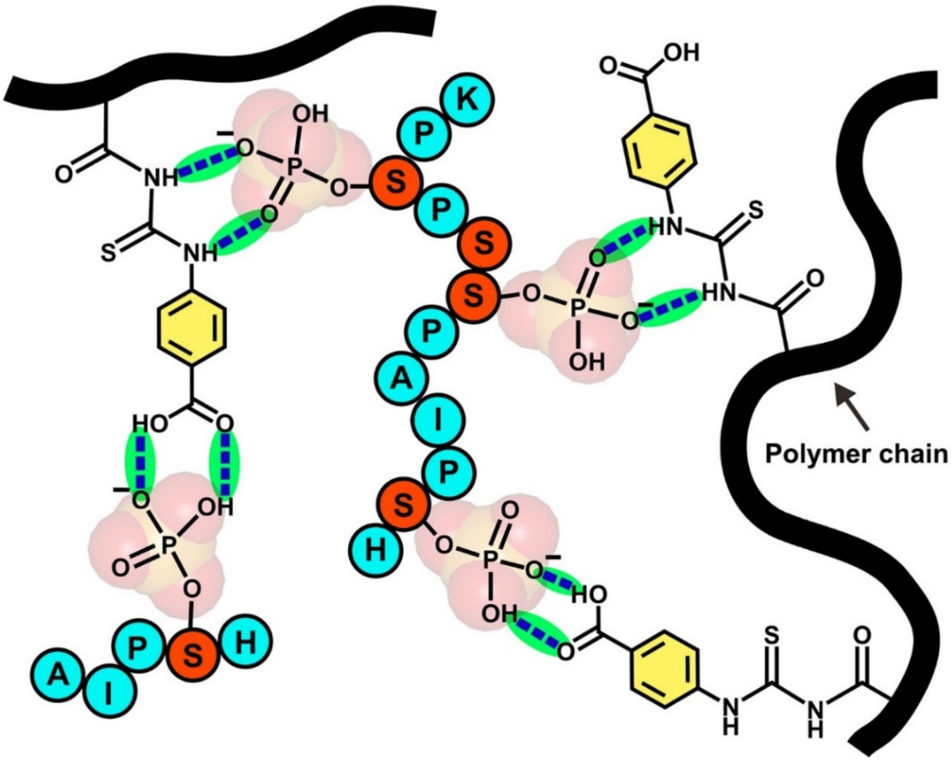


**Supplementary Figure 43 |** Illustration of possible binding mode of PNI-*co*-ATBA$_{0.2}$ with MPPs. In this system, PNIPAAm provides a flexible polymer main chain and ATBA functions as recognizing or binding unit for MPP. Through multiple hydrogen bonding interactions as indicated by blue dashed lines and green background, three ATBA units appended in the polymer chains could bind to a MPP simultaneously, which substantially improves the binding affinity of the polymer towards the MPP.

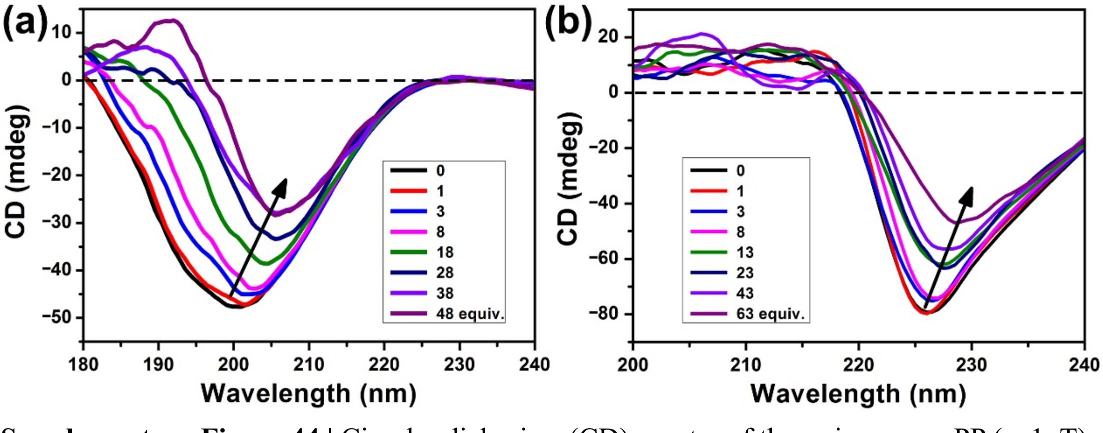


**Supplementary Figure 44 |** Circular dichroism (CD) spectra of threonine mono-PP (a, 1pT) or tri-PP (b, 3pT) with additions of various amounts of ATBA monomers in aqueous solution at 20 °C. The PP concentrations were $2 \times 10^{-4}$ mol·L$^{-1}$. Since ATBA was CD silence, this data demonstrated that ATBA could interact with both 1pT and 3pT, leading to remarkable decreases in the negative peaks (centered at 200 or 225 nm) of threonine PPs in the CD spectra.

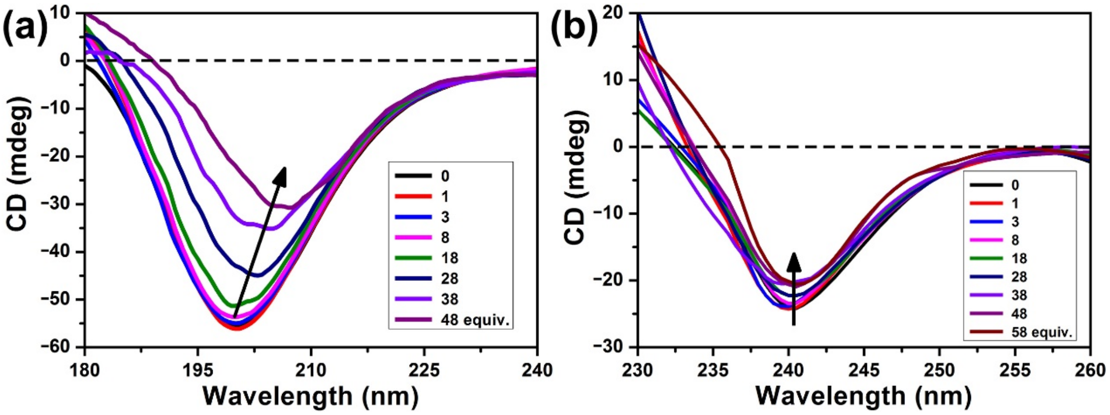


**Supplementary Figure 45 |** CD spectra of tyrosine mono-PP (a, 1pY) or tri-PP (b, 3pY) with additions of various amounts of ATBA monomers in aqueous solution at 20 °C. The PP concentrations were $2 \times 10^{-4}$ mol·L$^{-1}$. This data demonstrated that ATBA could interact with both 1pY and 3pY, leading to obvious decreases in the negative peaks (centered at 198 or 240 nm) of tyrosine PPs in the CD spectra.

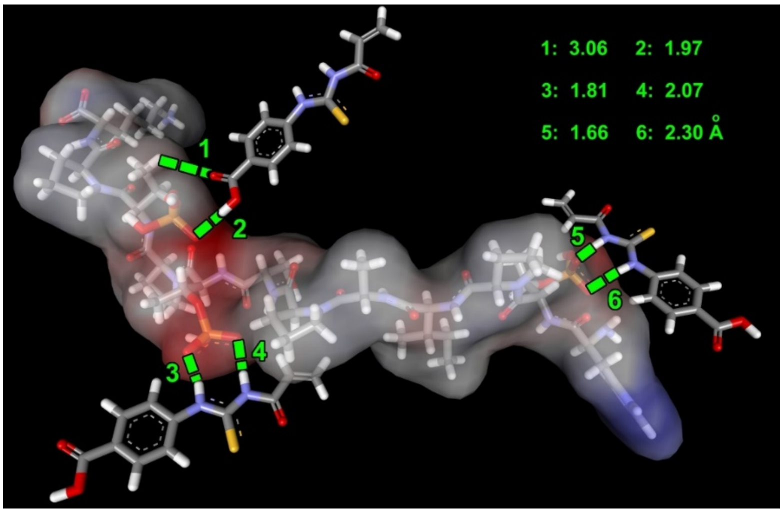


**Supplementary Figure 46 |** Possible binding model of threonine tri-PP (3pT) with three ATBA molecules, obtained from quantum chemistry calculation (Gaussian 2003, density function theory (DFT), at 3-21G level of theory). In this model, hydrogen bonding interactions between phosphate groups in 3pT and thiourea or carboxylic acid groups in ATBA are indicated by green dashed lines with different lengths. 3pT sequence: H(p-T)PIAPS(p-T)P(p-T)PK.

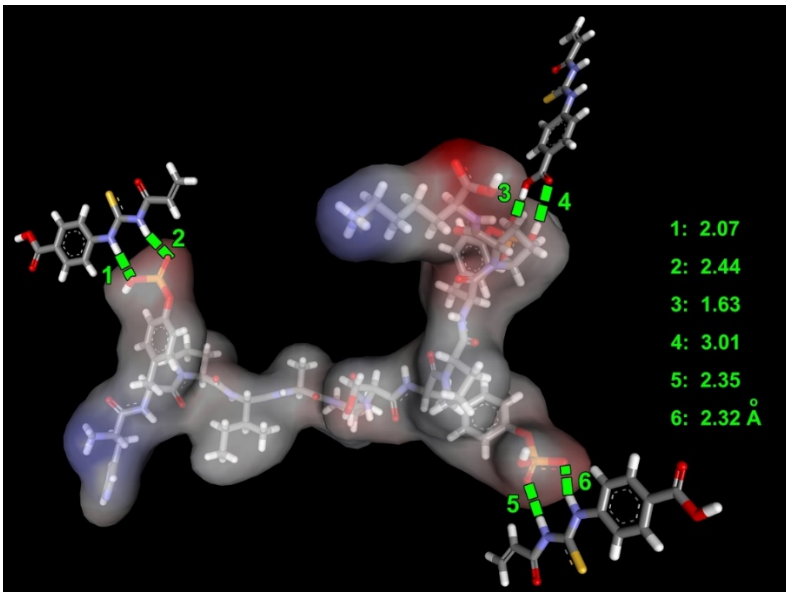


**Supplementary Figure 47 |** Possible binding model of tyrosine tri-PP (3pY) with three ATBA molecules, obtained from quantum chemistry calculation (Gaussian 2003, DFT, at 3-21G level of theory). In this model, hydrogen bonding interactions between phosphate groups in 3pY and thiourea or carboxylic acid groups in ATBA are indicated by green dashed lines with different lengths. 3pY sequence: H(p-Y)PIAPS(p-Y)P(p-Y)PK.

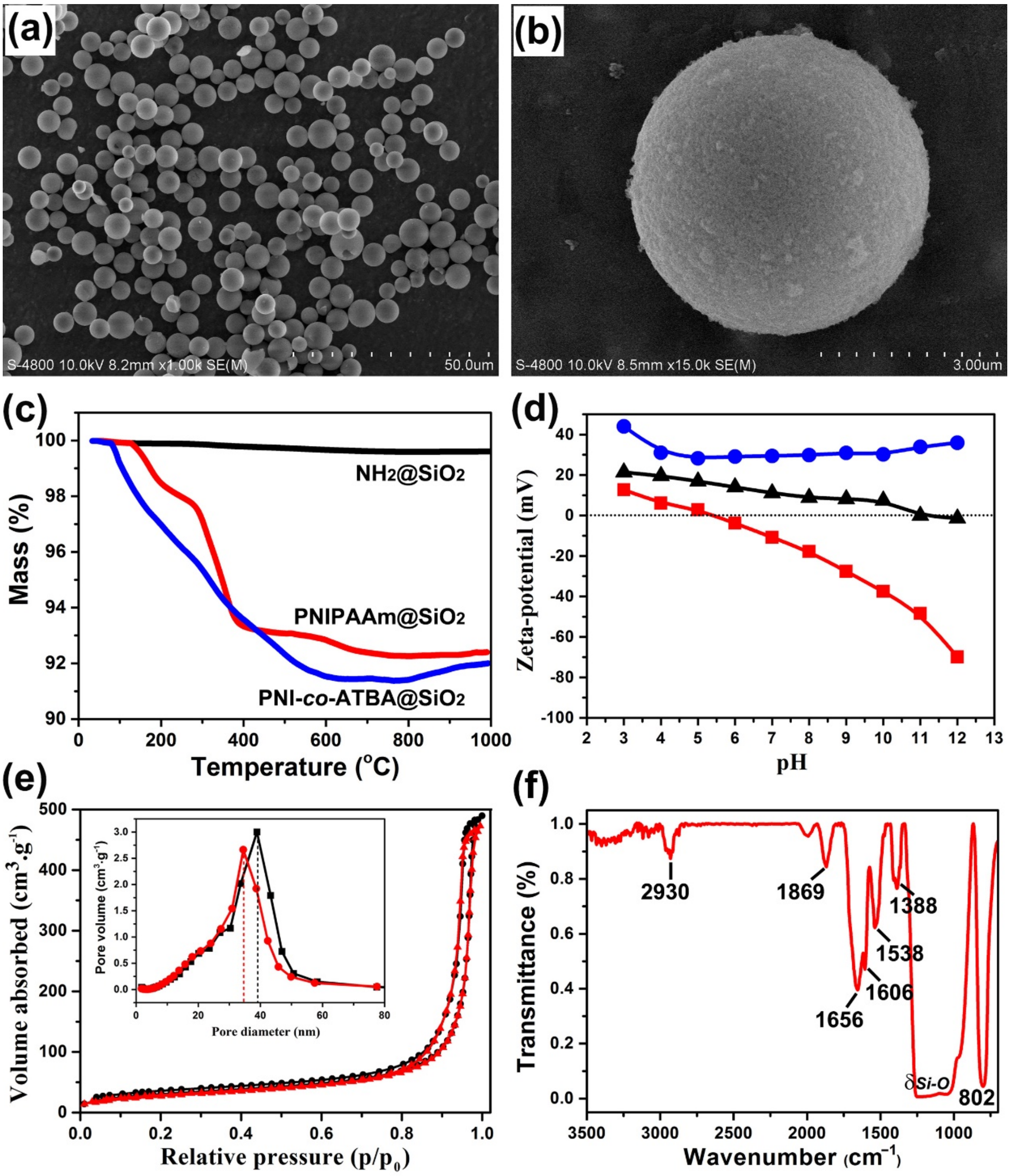


**Supplementary Figure 48 |** Scanning electrical microscopy (SEM) images of PNI-*co*-$ATBA_{0.2}$-modified silica gels. (a) Large-scaled image of silica gels; (b) magnified image of an individual silica microsphere with an average diameter of 5 μm; (c) thermal gravimetric analysis (TGA) curves of amino– (black), PNIPAAm– (blue) or PNI-*co*-$ATBA_{0.2}$ (red) modified silica gels; (d) Zeta potential change of amino– (●), PNIPAAm– (▲) or PNI-*co*-ATBA (■) modified silica gels with pH values; (e) BET adsorption isotherm curves of silica gels before (black) and after (red) modification with PNI-*co*-$ATBA_{0.2}$, the inset shows the corresponding pore size distribution, evidential decrease in the pore size indicated that PNI-*co*-$ATBA_{0.2}$ had been grafted into pores of silica gels; (f) FT-IR spectra of PNI-*co*-$ATBA_{0.2}$ modified silica gels, characteristic peaks of the copolymer: ($cm^{-1}$) 2930: $\nu s_{COOH}$; 1869: $\delta_{Ar\text{-}CO}$; 1656: $\delta_{CONH\ (amide\ I)}$; 1606: $\delta_{CONH\ (amide\ II)}$; 1538: $\delta_{CSNH}$; 1388: $\delta_{CH3\ or\ CCH3}$;

802: $\delta_{Ar\text{-}H}$.

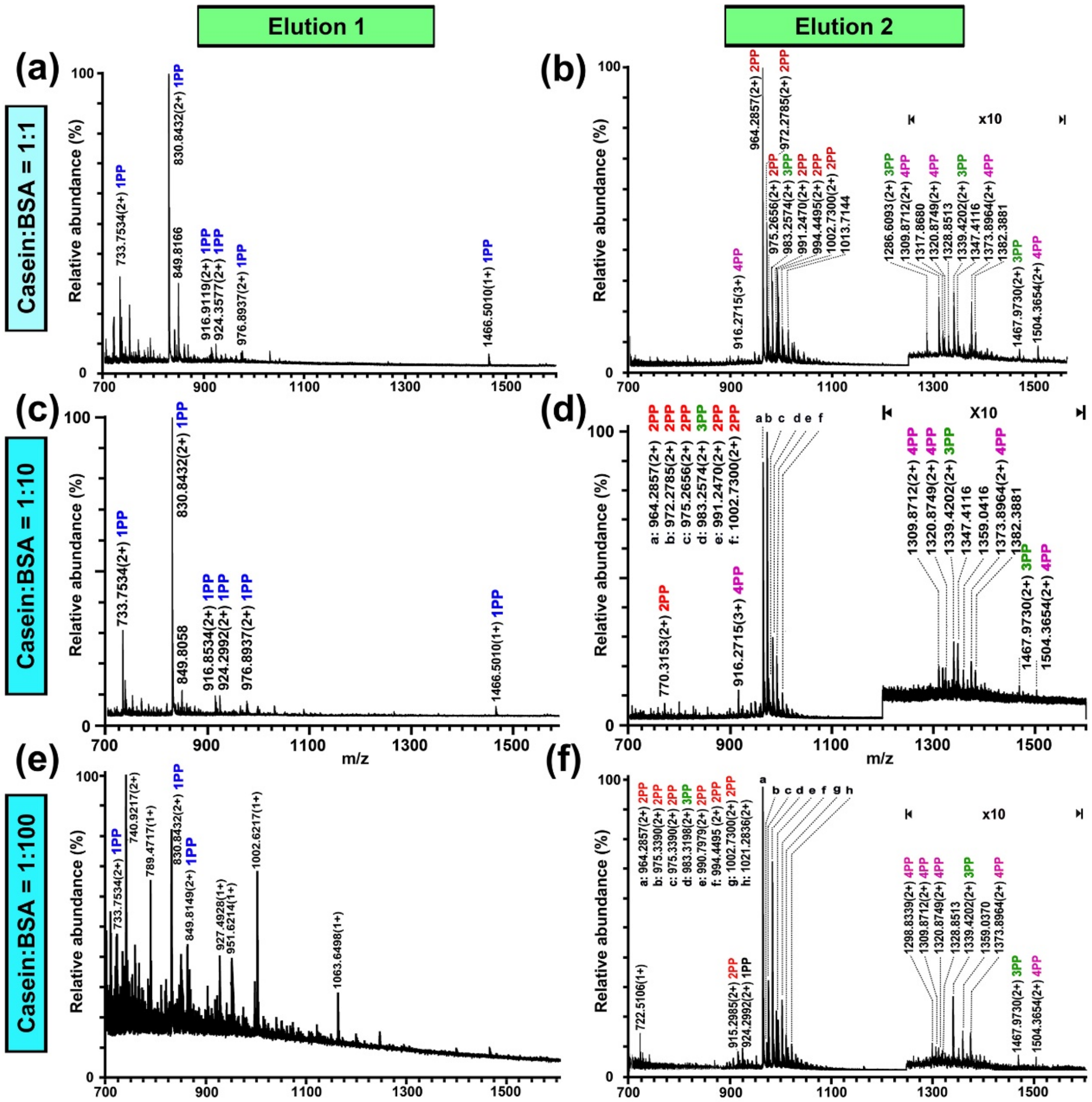


**Supplementary Figure 49 |** Nano-ESI-Q-TOF mass spectra of SPP (a, c, e) and MPP fractions (b, d, f) enriched with PNI-*co*-$ATBA_{0.2}@SiO_2$ from tryptic digests of α-casein and bovine serum albumin (BSA) at molar ratios of 1:1 (a, b), 1:10 (c, d) and 1:100 (e, f). The NMPs are labeled with their m/z values and PPs are marked with colors (blue: SPPs, red: 2PPs, green: 3PPs and purple: 4PPs) and the numbers of phosphorylation sites. The y-axis in each panel represents relative abundance in MS. The base peak (the tallest peak) of a particular ion is normalized to 100%. The other peaks in MS appear between 0–100% abundance. MS spectra and the corresponding PP signals indicated that SPPs and MPPs could be separated into two fractions through stepwise elution with $CH_3CN/H_2O$ solution by PNI-*co*-$ATBA_{0.2}@SiO_2$.

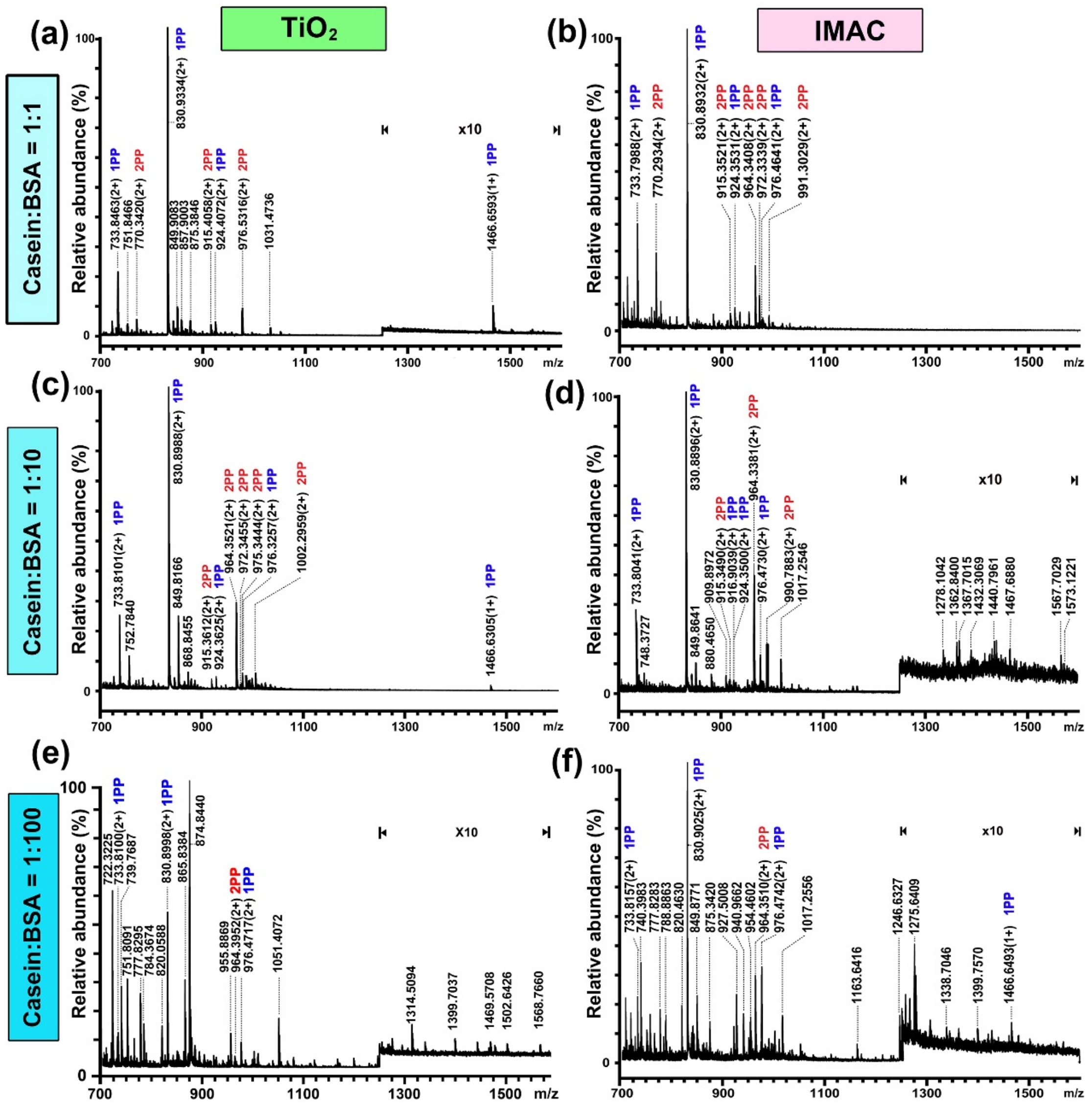


**Supplementary Figure 50 |** Nano-ESI-Q-TOF mass spectra of PPs enriched with commercially available $TiO_2$ (a, c, e) or IMAC materials (b, d, f) from tryptic digests of α-casein and BSA at molar ratios of 1:1 (a, b), 1:10 (c, d), and 1:100 (e, f). The NMPs are labeled with their m/z values and PPs are marked with colors (blue: SPPs, red: MPPs) and the numbers of phosphorylation sites. The y-axis in each panel represents relative abundance in MS. The base peak (the tallest peak) of a particular ion is normalized to 100%. The other peaks in MS appear between 0–100% abundance. Both $TiO_2$ and IMAC materials demonstrated high enrichment selectivity for 1PPs and 2PPs, even with the interference of 100-fold BSA. However, 3PP and 4PP signals were seldom detected by $TiO_2$ or IMAC materials.

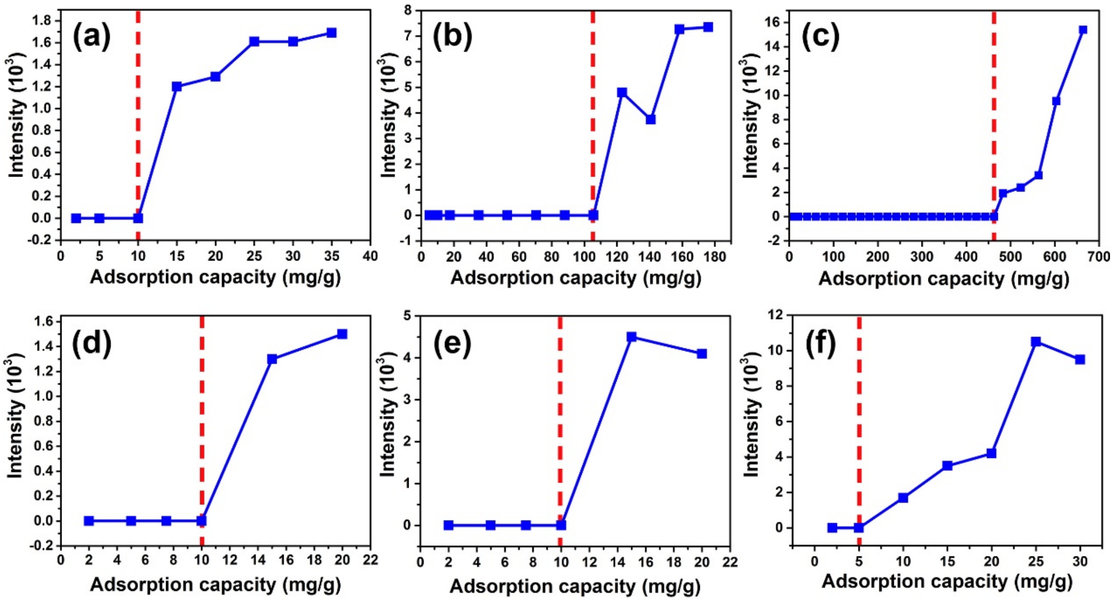


**Supplementary Figure 51 |** Determination of adsorption capacities of model serine di-PP (a, d), tri-PP (b, e) and tetra-PPs (c, f) on PNI-*co*-$ATBA_{0.2}$@$SiO_2$ (a, b, c) or commercially available $TiO_2$ (d, e, f) surfaces. Detailed measurement method is described in Methods section. The adsorption capacity was measured as the highest PP to adsorbing material ratio at which no PP signals were observed in the MS spectra, as indicated by red dashed lines. The optimized PP loading conditions: (a–c): 85% ACN/0.1% FA (pH = 2.59); (d–f): 80% $CH_3CN$ containing 1 M glycolic acid and 5% TFA.

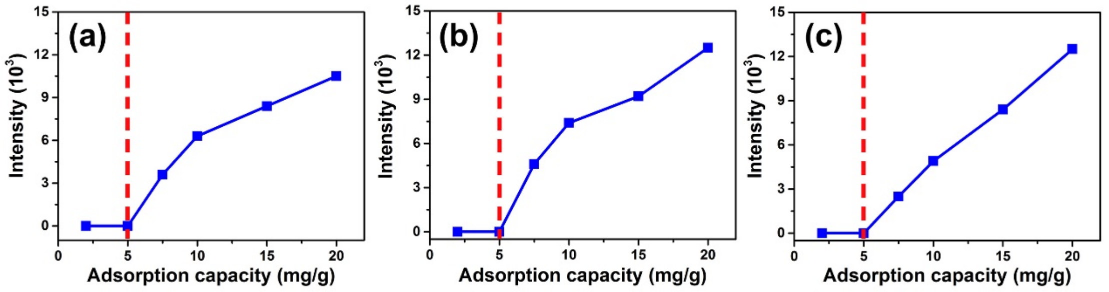


**Supplementary Figure 52 |** Determination of adsorption capacities of serine di-PP (a), tri-PP (b) and tetra-PPs (c) on PNI-*co*-$ATBA_{0.2}$@$SiO_2$. PP loading conditions: 75% $CH_3CN$/0.1% FA (pH = 2.59). The sharp decreases in adsorption capacities of the copolymer material towards 2pS, 3pS and 4pS, suggested the excellent tenability of material by altering the solvent polarity.

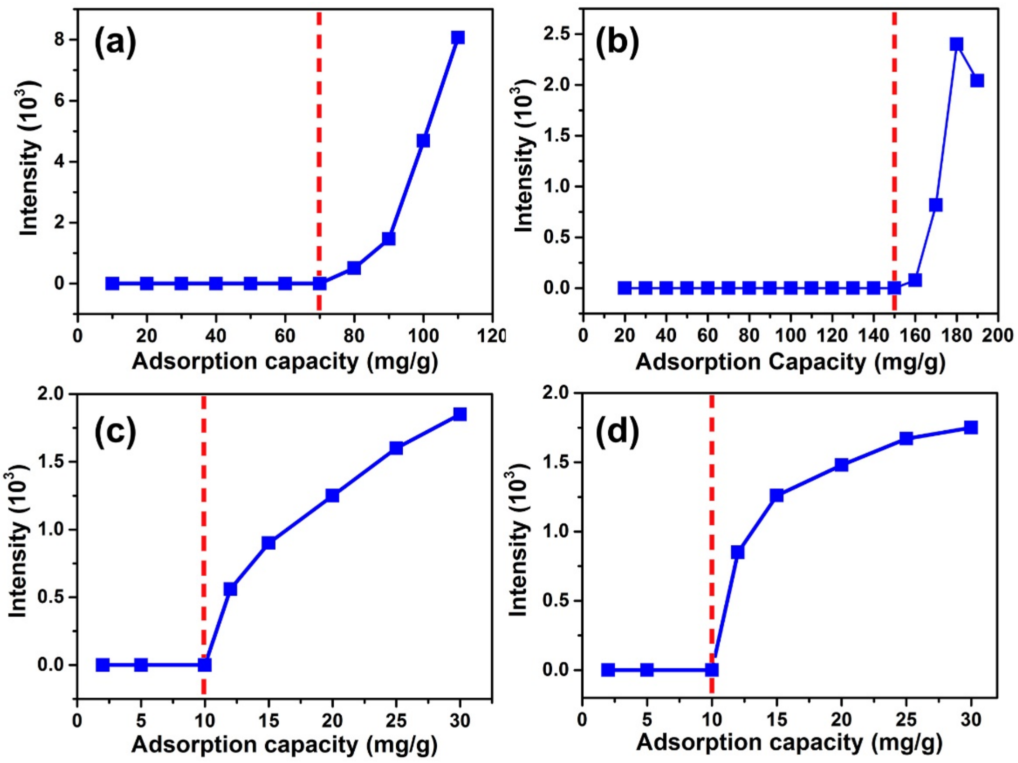


**Supplementary Figure 53 |** Determination of adsorption capacities of threonine (a, c, 3pT) or tyrosine (b, d, 3pY) tri-PPs on PNI-*co*-$ATBA_{0.2}$@$SiO_2$ (a, b) or commercially available $TiO_2$ (c, d) surfaces. The optimized 3pT or 3pY loading condition: (a, b): 85% ACN/0.1% FA (pH = 2.59); (c, d): 80% $CH_3CN$ containing 1 M glycolic acid and 5% TFA.

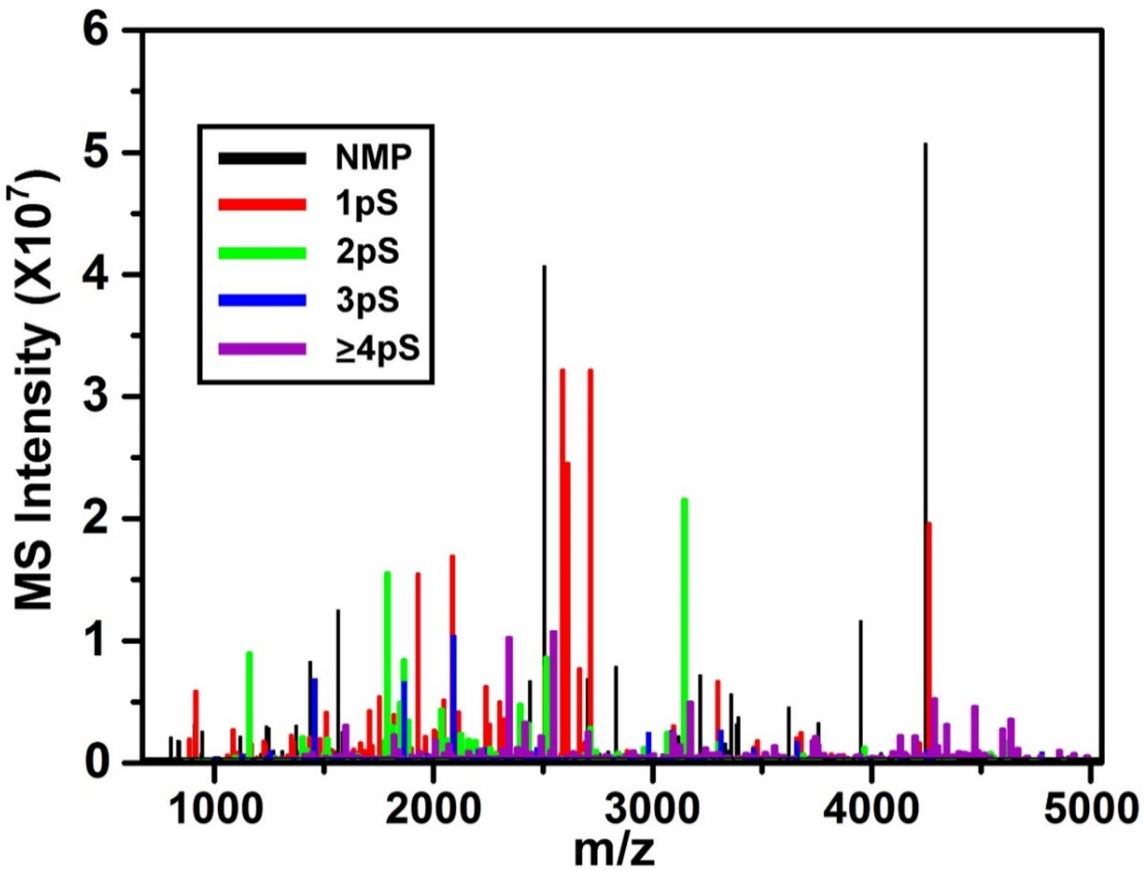


**Supplementary Figure 54 |** Distribution of the signal intensities for non-modified peptides (NMPs) and PPs with different numbers of phosphorylation sites. This result indicates that only a few NMPs with high signal intensities were observed by MS, the peptide sequences of the top 20 NMPs are listed in **Supplementary Table 6**, most of which have multiple Asp or Glu residues located right next to each other. Among PPs, the mono-PPs are the most abundant species, followed by di-PPs and tri-PPs. For the case of PPs with more than 4 phosphorylation sites, they show the lowest signal intensities among all identified peptides but could also be detected efficiently.

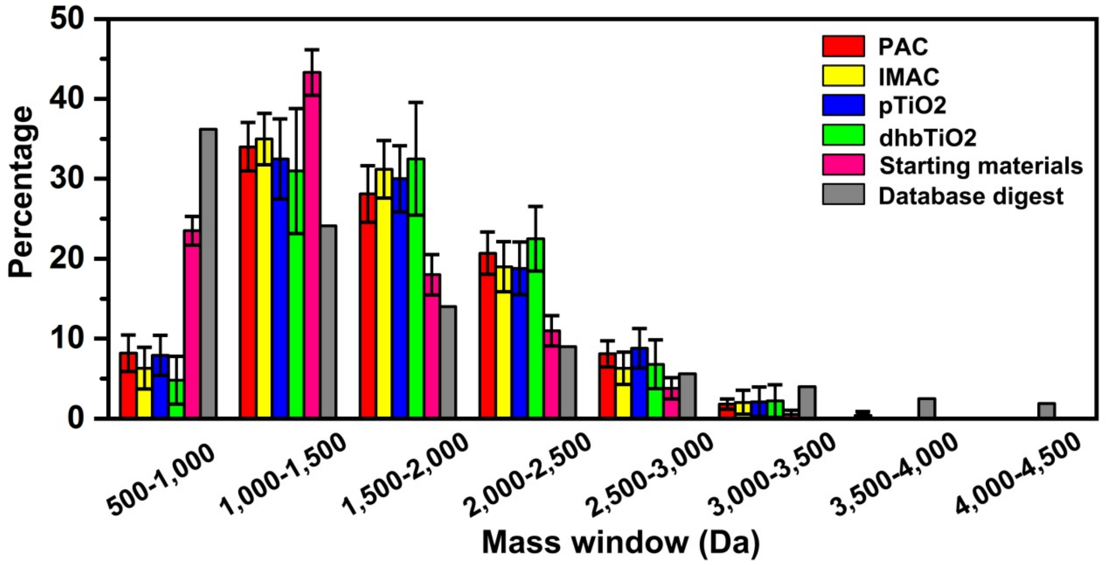


**Supplementary Figure 55 |** Distribution of molecular mass of PPs enriched with different methods. As a comparison, peptides derivated from a tryptic digest of the protein database are shown. The data were adapted from a classical reference [8] and used to represent the enrichment performance of mainstreamed materials or methods.

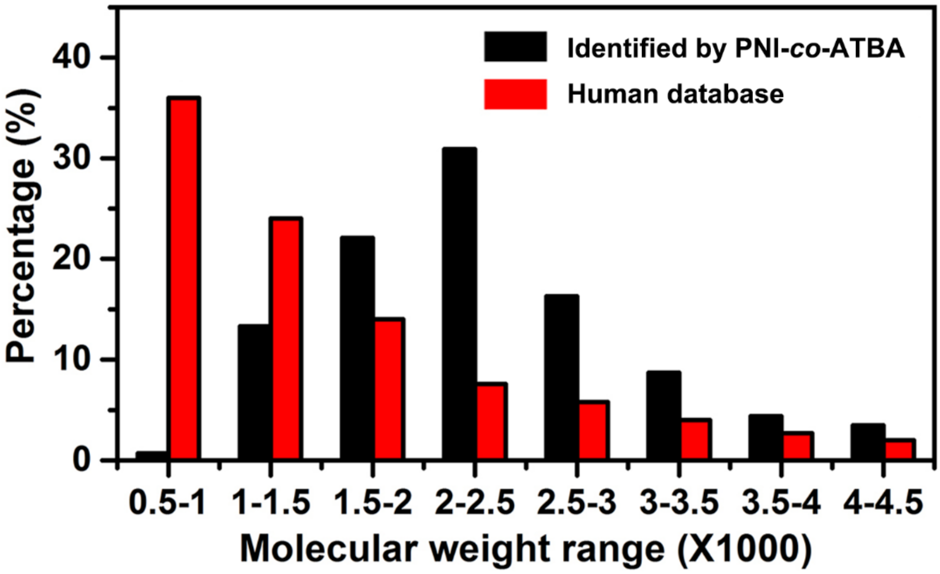


**Supplementary Figure 56 |** Comparison of molecular mass distribution of PPs collected by PNI-*co*-$ATBA_{0.2}$@$SiO_2$ and that collected in human database. This result indicated that our copolymer-based materials could enrich PPs with relatively larger molecular weight than theoretical tryptic digests of proteins in the human database.

**Supplementary Table 1 |** Association constants ($K_a$) of fluorescein-labeled thioureido-benzoic acid interacted with various anions in Tris-HCl buffer solution (10 mM, pH 7.4) at 20 °C.

| | $H_3PO_4$ | $H_2PO_4^-$ | $HPO_4^{2-}$ |
|---|---|---|---|
| $K_a$ ($L \cdot mol^{-1}$)[a,b] | [c] | 500 ± 150 | (5.34 ± 0.65) × $10^4$ |
| | $PO_4^{3-}$ | Benzene phosphate | |
| $K_a$ ($L \cdot mol^{-1}$)[a,b] | (4.01 ± 0.52) × $10^4$ | (7.80 ± 0.28) × $10^4$ | |
| | $CH_3COO^-$ | $HCOO^-$ | $Cl^-$ |
| $K_a$ ($L \cdot mol^{-1}$)[a,b] | (7.57 ± 0.82) × $10^3$ | (3.00 ± 0.35) × $10^3$ | (1.65 ± 0.17) × $10^3$ |

[a] $K_a$ values were obtained from fluorescence titration experiments based on the fluorescent intensity changes at the maximal emission peaks (approximately 514 nm);

[b] All error values were obtained by the results of nonlinear curve fitting, the correlation coefficient ($R$) is over 0.99. The nonlinear calculation equation is listed as below:

$$F = F_0 + \frac{F_{lim} - F_0}{2C_0}\{C_H + C_G + 1/K_a - [(C_H + C_G + 1/K_a)^2 - 4\, C_H \times C_G]^{1/2}\} \quad \textbf{eq. 1}$$

Where $F$ represents the fluorescent intensity, $F_0$ and $F_{lim}$ are the initial and ultimate fluorescent intensity, respectively, and $C_H$ and $C_G$ are the corresponding concentrations of host fluorescein-labeled thioureido-benzoic acid and anion guest, $C_0$ is the initial concentration of host.

[c] Reliable $K_a$ could not be obtained due to too small change in fluorescence spectra.

**Supplementary Table 2 |** Association constants ($K_a$) of fluorescein-labeled thioureido-benzoic acid interacted with various anions in $CH_3CN/H_2O$ (v/v: 80:20) Tris-HCl buffer solution (10 mM, pH 7.4) at 20 °C.

| | $H_3PO_4$ | $H_2PO_4^-$ | $HPO_4^{2-}$ |
|---|---|---|---|
| $K_a$ (L·mol$^{-1}$) [a] | 440 ± 55 [b] | 690 ± 78 [b] | (1.75 ± 0.19) × $10^4$ [b] |
| | $PO_4^{3-}$ | $CH_3COO^-$ | $HCOO^-$ |
| $K_a$ (L·mol$^{-1}$) [a] | (1.95 ± 0.22) × $10^4$ [b] | 388 ± 45 [b] | 282 ± 38 [b] |

[a] $K_a$ values were obtained from fluorescence titration experiments based on the fluorescent intensity changes at the maximal emission peaks (approximately 520 nm); Corresponding fluorescence spectra changes are shown in **Supplementary Figure 8**.

[b] All error values were obtained by the results of nonlinear curve fitting, the correlation coefficient ($R$) is over 0.99. The nonlinear calculation equation is listed as below:

$$F = F_0 + \frac{F_{\lim} - F_0}{2C_0}\{C_H + C_G + 1/K_a - [(C_H + C_G + 1/K_a)^2 - 4\, C_H \times C_G]^{1/2}\} \quad \textbf{eq. 1}$$

Where $F$ represents the fluorescent intensity, $F_0$ and $F_{\lim}$ are the initial and ultimate fluorescent intensity, respectively, and $C_H$ and $C_G$ are the corresponding concentrations of host fluorescein-labeled thioureido-benzoic acid and anion guest, $C_0$ is the initial concentration of host.

**Supplementary Table 3 |** Association constants ($K_a$) of fluorescein-labeled thioureido-benzoic acid interacted with various anions in dimethylsulfoxide (DMSO) at 20 °C.

| | $H_3PO_4$ | $H_2PO_4^-$ | $HPO_4^{2-}$ |
|---|---|---|---|
| $K_a$ ($L \cdot mol^{-1}$) [a] | [d] | 290 ± 58 [b] | (7.14 ± 0.41) × $10^5$ [c] |
| | $PO_4^{3-}$ | $CH_3COO^-$ | $HCOO^-$ |
| $K_a$ ($L \cdot mol^{-1}$) [a] | (2.70 ± 0.22) × $10^6$ [c] | 620 ± 115 [b] | (6.81 ± 0.31) × $10^3$ [b] |

[a] $K_a$ values were obtained from fluorescent titration experiments based on the fluorescent intensity changes at the maximal emission peaks (approximately 532 nm);

[b] All error values were obtained by the results of nonlinear curve fitting (**Eq. 1**), the correlation coefficient ($R$) is over 0.99.

[c] All error values were obtained by the results of linear curve fitting, the correlation coefficient ($R$) is over 0.99. The linear calculation equation is listed as below, which is suitable for multiple stoichiometry curve fitting.

$$\log[(F - F_0)/(F_\infty - F)] = n\log[G] + \log K_a \quad \textbf{Eq. 2}$$

Where $F$ represents the fluorescent intensity, $F_0$ and $F_\infty$ are the initial and ultimate fluorescent intensity, respectively, and $G$ are the corresponding concentrations of anion guest, n is the stoichiometry.

[d] Reliable $K_a$ could not be obtained due to too small change in fluorescence spectra.

**Supplementary Table 4 |** Association constants ($K_a$) of *N*-terminus fluorescein-labeled NMPs or serine mono-, di, tri, tetra-PPs (1pS—4pS) interacted with ATBA monomer under different pH conditions at 20 °C.

| Peptides | $K_a$ (L·mol$^{-1}$)[a,b] | | | |
|---|---|---|---|---|
| | pH 2.0[c] | pH 3.0[c] | pH 4.0[c] | pH 5.0[c] |
| **NMP 1** | 57 ± 11 | 79 ± 17 | (3.94 ± 0.39) × $10^3$ | 190 ± 48 |
| **NMP 2** | 41 ± 14 | 120 ± 12 | (4.73 ± 0.24) × $10^3$ | (1.58 ± 0.19) × $10^3$ |
| **1pS** | 75 ± 19 | 170 ± 10 | (5.21 ± 0.51) × $10^3$ | (3.54 ± 0.39) × $10^3$ |
| **2pS** | 50 ± 10 | (1.21 ± 0.16) × $10^3$ | (8.51 ± 0.95) × $10^3$ | (7.76 ± 0.86) × $10^3$ |
| **3pS** | 58 ± 17 | (7.51 ± 0.54) × $10^3$ | (1.50 ± 0.10) × $10^4$ | (1.25 ± 0.14) × $10^4$ |
| **4pS** | 53 ± 12 | (1.14 ± 0.14) × $10^4$ | (2.24 ± 0.06) × $10^4$ | (1.74 ± 0.09) × $10^4$ |

| Peptides | $K_a$ (L·mol$^{-1}$)[a,b] | | | |
|---|---|---|---|---|
| | pH 6.4[c] | pH 7.4[c] | pH 8.4[c] | pH 10.0[c] |
| **NMP 1** | 934 ± 66 | 150 ± 26 | (1.06 ± 0.17) × $10^3$ | 767 ± 108 |
| **NMP 2** | (1.34 ± 0.14) × $10^3$ | 32 ± 10 | (1.60 ± 0.18) × $10^3$ | 218 ± 30 |
| **1pS** | 737 ± 79 | 107 ± 19 | 342 ± 39 | 517 ± 68 |
| **2pS** | (1.64 ± 0.19) × $10^3$ | 72 ± 16 | 640 ± 135 | 870 ± 102 |
| **3pS** | (3.05 ± 0.20) × $10^3$ | 220 ± 45 | (1.55 ± 0.18) × $10^3$ | (2.79 ± 0.31) × $10^3$ |
| **4pS** | (6.86 ± 0.22) × $10^3$ | (4.64 ± 0.55) × $10^3$ | (3.99 ± 0.23) × $10^3$ | (2.93 ± 0.34) × $10^3$ |

[a] $K_a$ values were obtained from fluorescence titration experiments based on the fluorescent intensity changes at the maximal emission peaks (approximately 514 nm);

[b] All error values were obtained by the results of nonlinear curve fitting (**Eq. 1**), the correlation coefficient ($R$) is over 0.99;

[c] Buffer solutions: pH 2.0: maleate; pH 3.0: chloroacetate; pH 4.0: formate; pH 5.0 and pH 6.4: pyridine; pH 7.4, pH 8.4: Tris-HCl; pH 10.0: ethanolamine. The concentrations were 1.0 mmol·L$^{-1}$ for all these buffer solutions.

**Supplementary Table 5 |** Phosphorylated peptides identified from tryptic digest of bovine α-casein

| No. | Peptide sequence [a] a molecular weight (calcd.) | Phosphorylation sites | $[M+H]^+$ (Calcd.) | Found mass and charge states | Assignment |
|---|---|---|---|---|---|
| **1** | EQL**pS**T**pS**EENSK (1250.56) | 2 | 1411.53 | 706.24 (z = 2) | S2 [141-151] |
| **2** | TVDME**pS**TEVFTK (1385.64) | 1 | 1466.63 | 733.78 (z = 2) | S2 [153-164] |
| **3** | TVDME**pS**TEVFTK (1401.64) | 1 | 1482.63 | 741.78 (z = 2) | S2 [153-164] |
| **4** | EQL**pS**T**pS**EENSKK (1378.66) | 2 | 1539.63 | 770.27 (z = 2) | S2 [141-152] |
| **5** | TVDME**pS**TEVFTKK (1513.73) | 1 | 1594.72 | 797.84 (z = 2) | S2 [153-165] |
| **6** | VPQLEIVPN**pS**AEER (1579.82) | 1 | 1660.81 | 830.87 (z = 2)<br>554.25 (z = 3) | S1 [121-134] |
| **7** | YLGEYLIVPN**pS**AEER (1751.87) | 1 | 1832.86 | 916.90 (z = 2) | S2 [17-37] |
| **8** | DIGSE**pS**TEDQAMEDIK (1766.75) | 1 | 1847.74 | 924.33 (z = 2) | S1 [58-73] |
| **9** | DIG**pS**E**pS**TEDQAMEDIK (1766.75) | 2 | 1927.72 | 964.31 (z = 2) | S1 [58-73] |
| **10** | DIG**pS**E**pS**TEDQAMEDIK (1782.75) | 2 | 1943.72 | 972.32 (z = 2) | S1 [58-73] |
| **11** | YKVPQLEIVPN**pS**AEER (1870.98) | 1 | 1951.97 | 976.45 (z = 2)<br>651.29 (z = 3) | S1 [119-134] |
| **12** | QMEAE**pS**I**pSpSpS**EEIVPN**pS**VEQ (2192.99) | 5 | 2593.90 | 1296.91 (z = 2)<br>864.91 (z = 3) | S1 [74-93] |
| **13** | NTMEHV**pSpSpS**EESII**pS**QETYK (2298.03) | 4 | 2618.96 | 1309.93 (z = 2)<br>873.61 (z = 3) | S2 [17-36] |
| **14** | pyroQMEAE**pS**I**pSpSpS**EEIVPNSVEQK [b] (2321.08) | 4 | 2624.00 | 1312.45 (z = 2)<br>874.95 (z = 3) | S1 [74-94] |
| **15** | QMEAE**pS**I**pSpSpS**EEIVPNSVEQK (2321.08) | 4 | 2641.01 | 1320.95 (z = 2) | S1 [74-94] |
| **16** | NTMEHVS**pSpSpS**EESIISQETYKQ | 3 | 2666.98 | 1333.99 (z = 2)<br>889.67 (z = 3) | S2 [17-36] |
| **17** | VNEL**pS**KDIG**pS**E**pS**TEDQAMEDIK (2437.12) | 3 | 2678.07 | 1339.48 (z = 2)<br>893.30 (z = 3) | S1 [52-73] |
| **18** | NTMEHV**pSpSpS**EE**pS**IISQETYKQ (2426.09) | 4 | 2747.02 | 1373.95 (z = 2)<br>916.32 (z = 3) | S2 [17-37] |
| **19** | EKVNEL**pS**KDIG**pS**E**pS**TEDQAMEDIK (2694.25) | 3 | 2935.18 | 1468.08 (z = 2)<br>979.02 (z = 3) | S1 [50-73] |

| | | | | | |
|---|---|---|---|---|---|
| **20** | NANEEEYSIG**pSpSpS**EE**pS**AEVATE EVK (2687.16) | 4 | 3008.09 | 1504.49 (z = 2) 1003.32 (z = 3) | S2 [61-85] |

[a] Oxidized methionine are labeled with a box.

[b] This sequence corresponds to a loss of ammonia from the *N*-terminal glutamine residue condensing to form pyroglutamate.

**Supplementary Table 6 |** The top 20 non-modified peptides with high signal intensity co-eluted with MPPs, enriched by PNI-*co*-ATBA$_{0.2}$@$SiO_2$ from HeLa S3 cell lysate.

| Sequence | Mass |
|---|---|
| LAADEDDDDDDEEDDDEDDDDDDFDDEEAEEKAPVKK | 4245.5433 |
| AAAAAAAGDSDSWDADAFSVEDPVR | 2506.0884 |
| AAEDDEDDDVDTKK | 1564.6377 |
| VKLAADEDDDDDDEEDDDEDDDDDDFDDEEAEEK | 3949.3584 |
| AAEDDEDDDVDTK | 1436.5427 |
| VAAAPDEDLDGDDEDDAEDENNIDNR | 2831.1125 |
| ACAEDDDEEDEEEEEEEPDPDPEMEHV | 3217.1143 |
| ADDLDFETGDAGASATFPMQCSALR | 2703.1429 |
| YFQINQDEEEEEDED | 1930.7228 |
| ADDVDQQQTTNTVEEPLDLIR | 2441.1558 |
| INETDTFGPGDDDEIQFDDIGDDDEDIDDI | 3357.3328 |
| ADIDNKEQSELDQDLDDVEEVEEEETGEETK | 3621.5337 |
| ADIDVSGPSVDTDAPDLDIEGPEGK | 2511.15 |
| AENDVDNELLDYEDDEVETAAGGDGAEAPAKK | 3391.4699 |
| FLESGGQDGAGDDDDLEDLEEAEEPDMEEDDDQK | 3756.4388 |
| KKNEPEDEEEEEEEEDEDEEEEDEDEE | 3383.1976 |
| AGDSLLLDPCRR | 1371.6929 |
| DAEDDGEEEDD | 1237.3742 |
| AGGEEEDDDDEAAGGR | 1591.587 |
| DYEEVGVDSVEGEGEEEGEEY | 2347.8976 |
| AKDDDDDDDDDAEM | 1583.5053 |

**Supplementary Table 7 |** Average molecular weight of NMPs and PPs identified by PNI-*co*-$ATBA_{0.2}@SiO_2$ from tryptic digests of HeLa S3 cell lysate. This indicated that the average molecular weight of the PPs increased gradually with the numbers of phosphorylation sites in the peptides.

| | PPs identified by PNI-co-$ATBA_{0.2}@SiO_2$ | | | | | |
|---|---|---|---|---|---|---|
| | **NMPs** | **1 PPs** | **2 PPs** | **3 PPs** | **4 PPs** | **≥5 PPs** |
| Average molecular weight of NMPs and PPs ($g{\cdot}mol^{-1}$) | 1940 | 2026 | 2082 | 2196 | 2550 | 3059 |

**Supplementary Table 8 |** Comparison of serine (S), threonine (T) and tyrosine (Y) phosphorylation site ratios identified by PNI-*co*-$ATBA_{0.2}@SiO_2$ from tryptic digests of HeLa S3 cell lysate and phosphorylation site information collected in the human database.

| Samples | The percentages of S, T and Y phosphorylation sites | | |
|---|---|---|---|
| | **S** | **T** | **Y** |
| HeLa cell lysate | 67.7% | 22.5% | 9.8% |
| Human database | 89.5% | 10.0% | 0.5% |

Remarkably higher T and Y phosphorylation site proportions displayed the powerful enrichment capacities of our copolymer material towards these rare phosphorylated species.

**Supplementary Data 1 |** Unique phosphorylation sites identified from HeLa S3 cell lysate by PNI-*co*-$ATBA_{0.2}@SiO_2$.

This data is listed as an individual Excel file!

**Supplementary Data 2 |** Non-modified peptides identified from HeLa S3 cell lysate by PNI-*co*-$ATBA_{0.2}@SiO_2$, these peptides were co-eluted with phosphopeptides.

This data is listed as an individual Excel file!

## Supplementary References


1. Hui, C. M., Pietrasik, J., Schmitt, M., Mahoney, C., Choi, J., Bockstaller, M. R. & Matyjaszewski, K. Surface-initiated polymerization as an enabling tool for multifunctional (nano-)engineered hybrid materials. *Chem. Mater.* **26**, 745–762 (2014).

2. Pastor, A. & Martínez-Viviente, E. NMR spectroscopy in coordination supramolecular chemistry: A unique and powerful methodology. *Coord. Chem. Rev.* **252**, 2314–2345 (2008).

3. Beer, P. D. & Gale, P. A. Anion recognition and sensing: The state of the art and future persepctives. *Angew. Chem. Int. Ed*. **40**, 486–516 (2001).

4. Pu, L. Fluorescence of organic molecules in chiral recognition. *Chem. Rev.* **104**, 1687–1716 (2004).

5. Martinez-Manez, R. & Sancenon, F. Fluorogenic and chromogenic chemosensors and reagents for anions. *Chem. Rev*. **103**, 4419–4476 (2003).

6. Fielding, L. Determination of association constants (*K*a) from solution NMR data. *Tetrahedron* **56**, 6151–6170 (2000).

7. Liu, G. M. & Zhang, G. Z. QCM-D studies on polymer behavior at interfaces, Sharma, S. K. Eds. (Springer Heidelberg, New York, 2013).

8. Bodenmiller, B., Mueller, L. N., Mueller, M., Domon, B. & Aebersold, R. Reproducible isolation of distinct, overlapping segments of the phosphoproteome. *Nat. Methods* **4**, 231–237 (2007).
